# Phytochemical Investigation of *Equisetum arvense* and Evaluation of Their Anti-Inflammatory Potential in TNFα/INFγ-Stimulated Keratinocytes

**DOI:** 10.3390/ph16101478

**Published:** 2023-10-16

**Authors:** Se Yun Jeong, Hyung-Seok Yu, Moon-Jin Ra, Sang-Mi Jung, Jeong-Nam Yu, Jin-Chul Kim, Ki Hyun Kim

**Affiliations:** 1 School of Pharmacy, Sungkyunkwan University, Suwon 16419, Republic of Korea; dlawktkark@naver.com; 2 Natural Product Research Institute, Korea Institute of Science and Technology, Gangneung 25451, Republic of Korea; hsyu1207@kist.re.kr; 3 Hongcheon Institute of Medicinal Herb, Hongcheon-gun 25142, Republic of Korea; ramj90@himh.re.kr (M.-J.R.); sgmo77@naver.com (S.-M.J.); 4 Nakdonggang National Institute of Biological Resources, Sangju 37242, Republic of Korea; susia000@nnibr.re.kr; 5 Division of Bio-Medical Science and Technology, University of Science and Technology (UST), Daejeon 34113, Republic of Korea

**Keywords:** *Equisetum arvense*, phytochemicals, TNFα, IFNγ, dermatitis

## Abstract

*Equisetum arvense* L. (Equisetaceae), widely known as ‘horsetail’, is a perennial plant found extensively across Asia. Extracts of *E. arvense* have been used in traditional medicine, particularly for the treatment of inflammatory disorders. This study aimed to determine the phytochemical compounds in *E. arvense* ethanolic extract and their anti-inflammatory properties. Subsequently, we isolated and identified nine secondary metabolites, including kaempferol 3,7-di-*O*-*β*-D-glucopyranoside (**1**), icariside B_2_ (**2**), (*Z*)-3-hexenyl *β*-D-glucopyranoside (**3**), luteolin 5-*O*-*β*-D-glucopyranoside (**4**), 4-*O*-*β*-D-glucopyranosyl caffeic acid (**5**), clemastanin B (**6**), 4-*O*-caffeoylshikimic acid (**7**), (7*S*,8*S*)-*threo*-7,9,9′-trihydroxy-3,3′-dimethoxy-8-*O*-4′-neolignan-4-*O*-*β*-D-glucopyranoside (**8**), and 3-*O*-caffeoylshikimic acid (**9**). The chemical structures of the isolated compounds (**1–9**) were elucidated using HR-ESI-MS data, NMR spectra, and ECD data. Next, the anti-inflammatory effects of the isolates were evaluated in tumor necrosis factor (TNF)α/interferon (IFN)γ-induced HaCaT, a human keratinocyte cell line. Among the isolates, compound **3** showed the highest inhibitory effect on the expression of pro-inflammatory chemokines, followed by compounds **6** and **8**. Correspondingly, the preceding isolates inhibited TNFα/IFNγ-induced activation of pro-inflammatory transcription factors, signal transducer and activator of transcription 1, and nuclear factor-κB. Collectively, *E. arvense* could be employed for the development of prophylactic or therapeutic agents for improving dermatitis.

## 1. Introduction

Inflammation is one of the prominent protective responses of the host against external and internal stimuli, and it is pivotal for maintaining and recovering health [1]. However, an uncontrolled inflammatory response disrupts cellular homeostasis, leading to cell injury, tissue necrosis, and, ultimately, the development of inflammation-associated diseases, such as atopic dermatitis, psoriatic arthritis, inflammatory bowel diseases, and rheumatoid arthritis [2,3]. Chronic skin inflammation is typically characterized by impaired skin barrier function, accompanied by dermal tissue hyperplasia/hypertrophy and the infiltration of various pro-inflammatory cells, including macrophages, eosinophils, and mast cells [4]. Additionally, the complex expression of Th1 and Th2 cytokines, particularly tumor necrosis factor (TNF)α and interferon (IFN)γ, plays a primary role in the development of dermal lesions [5]. Moreover, numerous studies have demonstrated that aberrant expression of TNFα and IFNγ contributes to the development of allergic skin inflammation, leading to dermal hypertrophy and apoptosis of keratinocytes in atopic skin lesions [6,7].

Atopic dermatitis is a representative chronic inflammatory skin disorder, affecting approximately 15–20% of children and 2–4% of adults, particularly in developed countries, and its incidence continues to rise [8,9]. It is characterized by common symptoms such as edema, erythema, pruritus, rash, and skin hypersensitivity [10]. Accumulated evidence has expanded our understanding of the crucial role of keratinocytes in mediating the recruitment and infiltration of pro-inflammatory cells by releasing pro-inflammatory chemokines and cytokines, including regulated on activation, normal T-cell expressed and secreted (RANTES/CCL5), thymus and activation regulated chemokine (TARC/CCL17), interleukin (IL)-1β, and IL-6, thereby contributing to the development of atopic skin lesions [11]. Consequently, patients with atopic dermatitis often exhibit an aberrant increase in the infiltration of T cells, dendritic cells, mast cells, and markedly elevated serum IgE levels [12,13]. The current standard therapy for atopic dermatitis involves the application of antihistamines and steroids to alleviate inflammatory responses; however, long-term use of these therapies often leads to side effects such as hypotension and cutaneous atrophy [14]. Therefore, there is growing interest in natural bioactive compounds derived from plant materials for the development of therapeutic agents for the treatment of atopic dermatitis [15].

*Equisetum arvense* L. (Equisetaceae), widely known as ‘horsetail’, is a dense perennial plant extensively found across Canada, the USA, Europe, and Asia [16]. *E. arvense* has a history of traditional medicinal use for the treatment of various illnesses, including bladder disorders and tuberculosis [17]. Additionally, it is employed as a hemostatic agent to manage excessive menstruation, as well as pulmonary, gastric, and nasal hemorrhages [18]. Pharmacological studies have unveiled additional medicinal uses of *E. arvense*, such as its role as an anti-inflammatory and antioxidant agent [18]. For example, the extract of *E. arvense* disrupts the multifunctionality of immunocompetent cells and reduces the synthesis of TNFα and IFNγ, thereby providing anti-inflammatory activity [19]. This extract also leads to a reduction in JNK phosphorylation, a factor involved in immune responses. Quercetin 3-O-glucoside in *E. arvense* has been suggested to inhibit JNK phosphorylation, resulting in an anti-inflammatory effect [20]. Furthermore, when the extract of *E. arvense* was topically applied in a rat periodontitis model, it effectively inhibited bone loss by suppressing osteoclast formation triggered by several inflammatory cytokines, including TNFα, IL-1β, and IL-6. Quercetin, present in *E. arvense*, has demonstrated the ability to inhibit osteoclast formation, proliferation, and maturation. Additionally, ursolic acid, another compound found in *E. arvense*, has been observed to promote osteoblast differentiation and mineralization [21]. The major ingredients in *E. arvense*, such as sterols (β-sitosterol and campesterol), ascorbic acid, phenolic acids (cinnamic acids and caffeic acid), flavonoids, and styrylpyrones, were reported to be involved in its therapeutic activity [22]. Recent studies have shown that *E. arvense* extract, rich in phenolic compounds, effectively inhibits *Aspergillus* and *Fusarium* species [23]. The ethanol extract of *E. arvense* exhibited significant antimicrobial and antioxidant activity, which is related to its high total phenolic content [24]. Furthermore, the presence of the glycosidic flavonoid isoquercitrin in *E. arvense* extract partly mediated its anti-inflammatory activity in vivo [25].

As part of ongoing research aimed at identifying bioactive natural metabolites from various natural sources [26,27,28,29,30], phytochemical analysis of the ethanol extract of the aerial parts of *E. arvense* was conducted using liquid chromatography-mass spectrometry (LC/MS). The isolated compounds were elucidated through data obtained from nuclear magnetic resonance (NMR) experiments and high-resolution electrospray ionization-mass spectrometry (HR-ESI-MS). The anti-inflammatory capacity of these isolated compounds was assessed in the TNFα/IFNγ-induced HaCaT cell line, a human keratinocyte line, to determine their biological activity. In this study, we present the isolation and structural determination of compounds **1**–**9**, as well as the evaluation of their anti-inflammatory effects in TNFα/IFNγ-induced HaCaT cells.

## 2. Results and Discussion

### 2.1. Isolation and Identification of the Compounds

**Figure 1:**
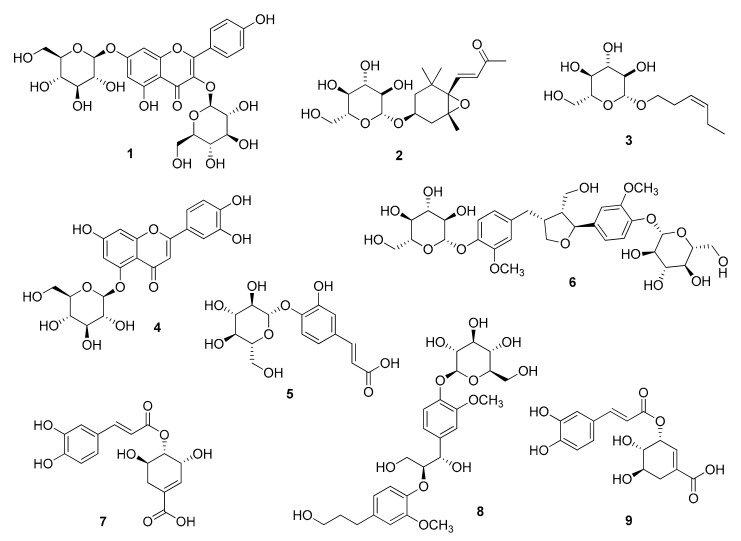
Chemical structures of compounds **1**–**9**.

**Figure 2:**
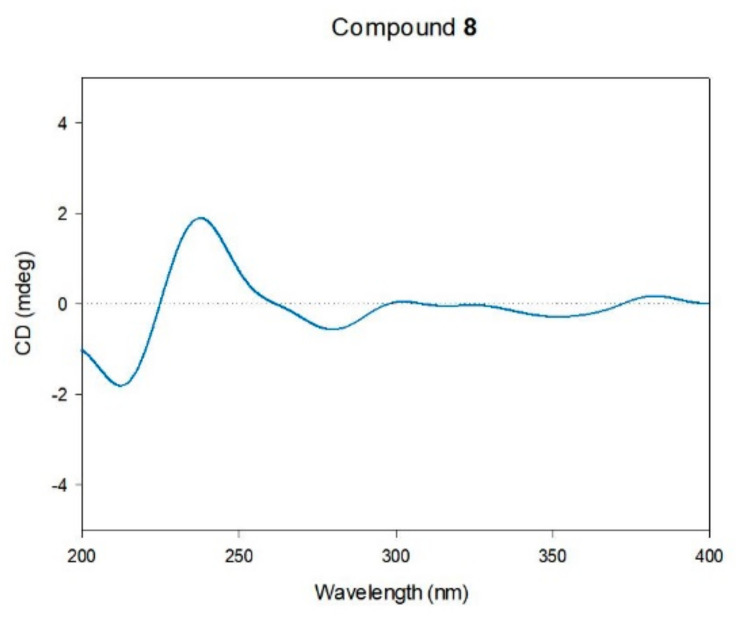
Experimental ECD of compound **8**.

To isolate bioactive compounds from *E. arvense*, we conducted LC/MS-guided isolation of the ethanol (EtOH) extract from the aerial parts of *E. arvense*. Initially, we identified that a significant portion of the metabolites consisted of sugars and glycosidic flavonoids by matching their mass and UV absorption spectra with our in-house libraries. We performed reversed-phase Sep-Pak chromatography to reduce the lipid content of the EtOH extract. Subsequently, we employed open-column chromatography with Diaion HP-20 using a mobile phase of distilled water followed by methanol (MeOH) to eliminate sugars. The resultant extract was fractionated into four fractions (M1–M4) using medium-pressure liquid chromatography (MPLC), and we confirmed that the major glycosidic flavonoids were present in fractions M2 and M3 through LC/MS analysis. Further isolation was carried out via semi-preparative HPLC for fractions M2 and M3, resulting in the isolation of nine metabolites, including glycosidic metabolites and caffeoylshikimic acids (Figure 1). The isolated compounds were identified as kaempferol 3,7-di-*O*-*β*-D-glucopyranoside (**1**) [31], icariside B_2_ (**2**) [32], (*Z*)-3-hexenyl *β*-D-glucopyranoside (**3**) [33], luteolin 5-*O*-*β*-D-glucopyranoside (**4**) [34], 4-*O*-*β*-D-glucopyranosyl caffeic acid (**5**) [35], clemastanin B (**6**) [36], 4-*O*-caffeoylshikimic acid (**7**) [37], (7*S*,8*S*)-*threo*-7,9,9′-trihydroxy-3,3′-dimethoxy-8-*O*-4′-neolignan-4-*O*-*β*-D-glucopyranoside (**8**) [38], 3-*O*-caffeoylshikimic acid (**9**) [39]. These identifications were based on the comparison of their NMR spectra with reported data and HR-ESI-MS analysis (Figures S1–S18). However, determining the absolute configuration of *erythro*- or *threo*-form in neolignans proved challenging based solely on the analysis of NMR data. Consequently, we confirmed the absolute configuration of compound **8** through electronic circular dichroism (ECD), which matched previously reported data for the related compound with the (7*S*,8*S*)-form [38] (Figure 2). Compounds **1** and **4**, flavonoid glycosides, have previously been reported as constituents of *E. arvense* [40]. Additionally, luteolin 5-*O*-*β*-D-glucopyranoside (**4**) has been discovered in *E. giganteum*, icariside B_2_ (**2**) in *E. sylvaticum*, and (*Z*)-3-hexenyl *β*-D-glucopyranoside (**3**) in *E. debile*, as reported in prior studies [41,42,43]. While various flavonoid glucosides, phenolic acid derivatives, terpenoids, fatty acids, lignan glycosides, and caffeic acid derivatives have already been reported in the *Equisetum* genus, compounds **5**–**9** have been newly identified in the *Equisetum* genus, marking a significant discovery [44].

### 2.2. Cell Viability Assessment

**Figure 3:**
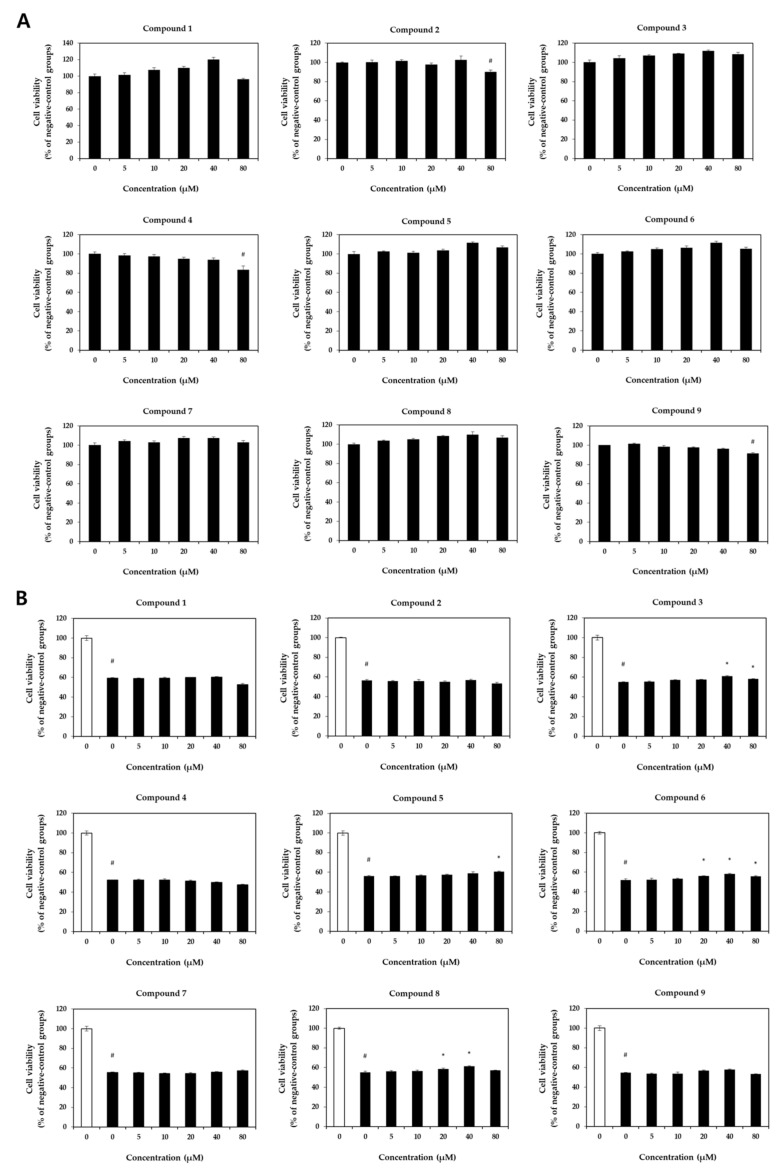
Effect of compounds on viability of HaCaT cells. The cells were treated with different concentrations of each compound (0–80 μM) and stimulated with or without TNFα/IFNγ. The cell viability was evaluated using an MTT assay. (**A**) Cell viability without TNFα/IFNγ stimulation. (**B**) cell viability with TNFα/IFNγ stimulation (□, un-treated negative-control groups; ■, TNFα/IFNγ-treated groups). Differences among the multiple groups were determined by ANOVA, followed by Tukey’s post-hoc test. # *p* < 0.05, compared to negative-group; * *p* < 0.05, compared to positive-group.

We conducted a dose-dependent cytotoxicity assessment in HaCaT cells using an MTT assay (Figure 3A,B). Treatment with the isolated compounds did not exhibit cytotoxicity up to a concentration of 40 μM (Figure 3A). In most of the compounds, cell viability was decreased in the 80 μM-treated groups compared to the 40 μM-treated groups. Particularly, compounds **2**, **4**, and **9** exhibited cytotoxicity at the 80 μM concentration. Compared to the negative control groups, stimulation with TNFα/IFNγ reduced cell viability to 56.38 ± 1.65% of the initial cell viability. However, the TNFα/IFNγ-induced reduction in cell viability was improved with the treatment of compounds, especially in groups treated with compounds **3**, **6**, and **8** (Figure 3B). Following the preceding results, a comparison of the anti-inflammatory effects of each compound was performed at a concentration of 40 μM, and the cytotoxic effect of each compound was not considered in subsequent experiments.

### 2.3. Expression of Pro-Inflammatory Chemokines and Cytokines

HaCaT cells stimulated with TNFα/IFNγ represent a well-established cellular model for screening potential candidates for the treatment of atopic skin lesions [45]. Stimulation with TNFα and IFNγ activates keratinocytes, leading to the overexpression of inflammation-associated mediators such as CCL17, CCL5, IL-1β, IL-6, and C-X-C motif chemokine ligand (CXCL)8. This response, in turn, promotes the infiltration of pro-inflammatory cells, ultimately resulting in the development of atopic dermatitis [9,13]. Consistent with previous findings, the overexpression of these chemokines and cytokines, along with an aberrantly increased infiltration of pro-inflammatory cells and markedly elevated serum IgE levels, has been observed in dermal tissues of patients with atopic dermatitis [12,13,46].

**Figure 4:**
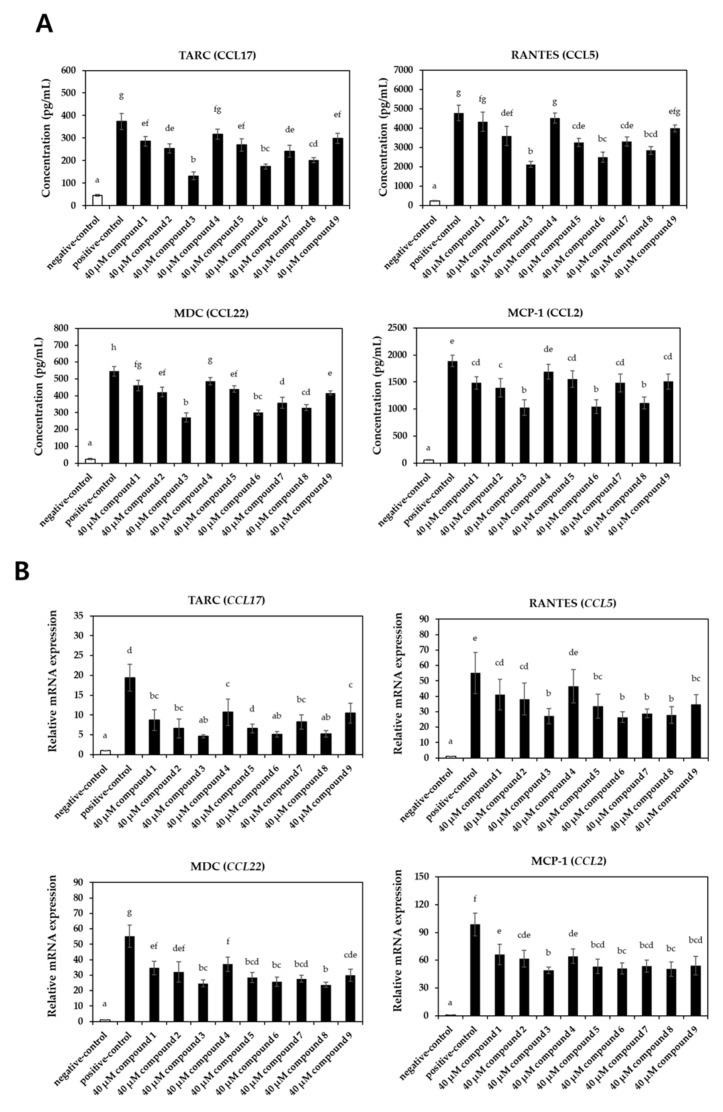
Effect of compounds on the expression levels of pro-inflammatory chemokines in TNFα/IFNγ-induced HaCaT cells. The cells were treated with 40 μM of each compound and stimulated with TNFα/IFNγ for 20 h (□, un-treated negative-control groups; ■, TNFα/IFNγ-treated groups)**.** (**A**) The production of chemokines was evaluated with commercial ELISA kits. (**B**) The relative mRNA expression levels of chemokines were investigated with qRT-PCR analysis. Values labeled with different letters (a–h) are significantly different (*p* < 0.05). Differences among the multiple groups were determined by ANOVA, followed by Tukey’s post-hoc test.

**Figure 5:**
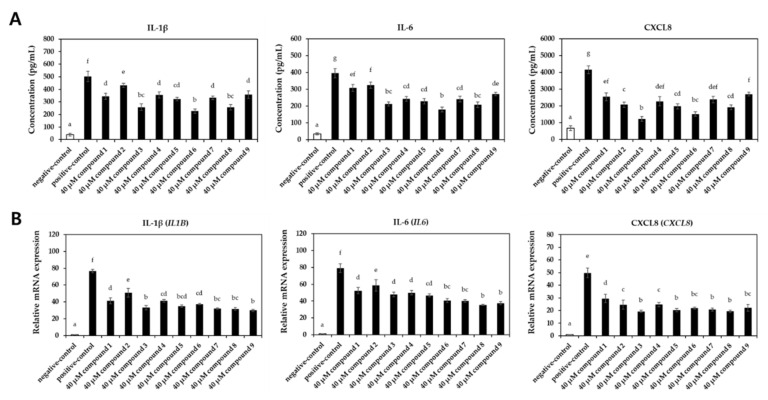
Effect of compounds on the expression levels of pro-inflammatory cytokines in TNFα/IFNγ-induced HaCaT cells. The cells were treated with 40 μM of each compound and stimulated with TNFα/IFNγ for 20 h (□, un-treated negative-control groups; ■, TNFα/IFNγ-treated groups). (**A**) The production of cytokines was assessed with commercial ELISA kits. (**B**) The relative mRNA expression levels of cytokines were determined with qRT-PCR analysis. Values labeled with different letters (a–g) are significantly different (*p* < 0.05). Differences among the multiple groups were determined by ANOVA, followed by Tukey’s post-hoc test.

The effects of the isolated compounds on the expression levels of pro-inflammatory chemokines and cytokines were assessed upon stimulation with TNFα/IFNγ to evaluate the anti-inflammatory potential (Figure 4 and Figure 5). Treatment with TNFα/IFNγ significantly increased the expression levels of chemokines (TARC, RANTES, MDC, and MCP-1) at both the protein (Figure 4A) and mRNA levels (Figure 4B). Notably, compounds **3**, **6**, and **8** significantly reduced the expression levels of these chemokines. Similarly, treatment with compounds **3**, **6**, and **8** significantly suppressed the TNFα/IFNγ-induced induction of cytokines (IL-1β, IL-6, and CXCL8) at both the protein (Figure 5A) and transcription levels (Figure 5B).

Pro-inflammatory chemokines and cytokines secreted by keratinocytes, such as TARC, MDC, IL-4, IL-6, and CXCL8, have been implicated in the development of skin inflammation and allergic reactions, as well as in the amplification of the overall immune response [47]. Therefore, inhibiting the overexpression of these pro-inflammatory mediators holds promise as a prophylactic and therapeutic strategy for atopy and atopic dermatitis. For example, ophiopogonin D, a steroidal glycoside derived from *Ophiopogon japonicus*, mitigated TNFα-induced production of IL-β and IL-6 in keratinocytes, which correlated with the attenuation of atopic dermal lesions in BALB/c mice following 2,4-dinitrochlorobenzene (DNCB) stimulation [48]. Similarly, the suppression of pro-inflammatory chemokines and cytokines, including CCL17, CCL5, CCL22, and IL-6, upon treatment with phytochemical derivatives, has been shown to inhibit the development of atopic skin lesions [6,49]. In this study, the isolated compounds from *E. arvense* demonstrated the ability to inhibit the production and secretion of pro-inflammatory chemokines and cytokines, suggesting that *E. arvense* may have the potential to contribute to the improvement of atopic dermatitis.

### 2.4. Activation of Pro-Inflammatory Transcription Factors

**Figure 6:**
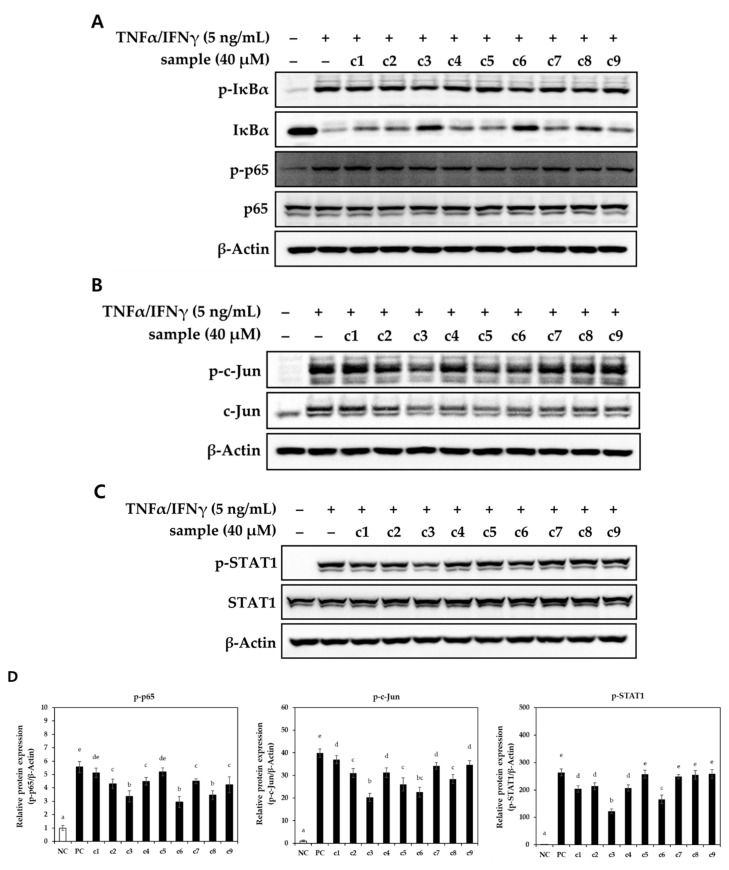
Effect of compounds on the protein expression levels of inflammation-associated protein in TNFα/IFNγ-induced HaCaT cells. Following the starvation, cells were treated with 40 μM of each compound and stimulated with TNFα/IFNγ for 10 min. The c1–c9 represent compounds 1–9. Phosphorylated (**A**) IκBα, p65, (**B**) c-Jun, and (**C**) STAT1 were visualized by using western blotting. (**D**) The relative protein expression levels of phosphorylated p65, c-Jun, and STAT1 were calculated. The representative blot was presented (*n* = 3). Values labeled with different letters (a–e) are significantly different (*p* < 0.05). Differences among the multiple groups were determined by ANOVA, followed by Tukey’s post-hoc test.

NF-κB, AP-1 (c-Jun), and STAT1 are pivotal pro-inflammatory transcription factors that become activated in response to various stimuli, such as lipopolysaccharide, TNFα, and IFNγ. Upon stimulation, these transcription factors translocate from the cytoplasm to the nucleus, where they contribute to the expression of various pro-inflammatory genes [48,50]. Previous studies have shown that the promoters of chemokines and cytokines, including CCL17, CCL2, CCL22, and IL-6, contain NF-κB, AP-1, and STAT-binding sites, indicating that the regulation of these transcription factors can modulate pro-inflammatory mediators [50,51]. In the current study, we demonstrated that compounds **3**, **6**, and **8** significantly suppressed representative pro-inflammatory transcription factors upon TNFα/IFNγ stimulation (Figure 6A,B), which correlated with reduced expression levels of pro-inflammatory chemokines and cytokines. Notably, treatment with compounds **3**, **6**, and **8** inhibited the degradation of the IκBα/NF-κB complex, suggesting suppression of the nuclear translocation of NF-κB. The alleviation of c-Jun activation by compounds **3**, **6**, and **8** may have contributed to their protective effect against TNFα/IFNγ-induced cytotoxicity, as there is a known correlation between JNK/AP-1 signaling pathways and cell apoptosis [1]. In addition, compounds **3** and **6** showed a significant inhibitory effect on TNFα/IFNγ-induced phosphorylation of STAT1. Our results align with previous studies that have shown that inhibiting the activation of transcription factors, including NF-κB, AP-1 (c-Jun), and STAT1, leads to a reduction in the expression of pro-inflammatory chemokines and cytokines, ultimately improving atopic skin lesions [6,7,8,48,49,50,51]. In summary, our results provide evidence that the inhibitory effect of the compounds **3**, **6**, and **8** on the expression of pro-inflammatory chemokines and cytokines is mediated by the blockade of NF-κB, AP-1, and STAT1 signaling pathways.

The active compound, (*Z*)-3-hexenyl *β*-D-glucopyranoside (**3**) was previously reported as a potent anti-inflammatory metabolite from *Miliusa balansae*, inhibiting nitric oxide production in lipopolysaccharide-stimulated murine RAW 264.7 macrophage cells with inhibition values of 91.8 ± 2.7% (20.0 μM) [52]. Additionally, clemastanin B (**6**), derived from the roots and rhizomes of *Valeriana amurensis*, exhibited neuroprotective effects on PC12 cells, countering neurotoxicity induced by amyloid β1-42 in a previous study [53]. Furthermore, the aglycone of compound **6** from *Ginkgo biloba* demonstrated anti-inflammatory activity by inhibiting TNF-α-induced NF-κB transcriptional activity in HepG2 cells and activating transcriptional activity of peroxisome proliferator-activated receptors (PPARs) in a dose-dependent manner [54]. However, other lignans with a different position of glucose compared to compound **6** and the aglycone of compound **8** did not exhibit significant anti-inflammatory properties [55]. These outcomes support the idea that the presence and position of glucose within lignans can significantly affect their anti-inflammatory activity [56,57].

## 3. Materials and Methods

### 3.1. General Experimental Procedure

Optical rotations were measured using a Jasco P-2000 polarimeter (Jasco, Easton, MD, USA). Electronic circular dichroism spectra were measured on a Jasco J-1500 spectropolarimeter (Jasco). Nuclear magnetic resonance (NMR) spectra were recorded with a Bruker AVANCE III HD 850 NMR spectrometer, equipped with a 5 mm TCI CryoProbe operating at 850 MHz (^1^H) and 212.5 MHz (^13^C) (Bruker, Karlsruhe, Germany). Chemical shifts were reported in ppm (δ) for both ^1^H and ^13^C NMR analyses and were referenced to the solvent peaks of CD_3_OD at 3.310 ppm for ^1^H, 49.000 ppm for ^13^C, and DMSO-*d*_6_ at 2.500 ppm for ^1^H (Cambridge Isotope Laboratories, Inc., 50 Frontage Rd, Andover, MA, USA). LC/MS analysis was performed on an Agilent 1200 Series HPLC system equipped with a diode array detector and a 6130 Series ESI mass spectrometer (Agilent Technologies, Santa Clara, CA, USA). An analytical Kinetex C18 column (100 × 2.1 mm, 5 μm; flow rate: 0.3 mL/min; Phenomenex, Torrance, CA, USA) was used for the analysis. All HR-ESI-MS data were obtained with an Agilent 6545 Q-TOF LC/MS spectrometer (Agilent Technologies) using an analytical ZORBAX Eclipse Plus C18 column (50 × 2.1 mm, 1.8 μm; flow rate: 0.3 mL/min; Agilent Technologies). Sep-Pak solid-phase extraction was conducted using Strata C18-E (55 μm, 70Å, 2 g /12 mL giga tubes, Phenomenex). Column chromatography was carried out with Diaion HP-20 (IONTEC, Sungnam, Republic of Korea). MPLC was performed using a Yamazen Smart Flash AKROS (Yamazen Corporation, Osaka, Japan) with a Universal column (16.5 × 3.0 cm, 40 μm; flow rate: 10 mL/min; Yamazen Corporation). Semi-preparative HPLC was performed using a Waters 1525 Binary HPLC pump with a Waters 996 Photodiode Array Detector (Waters Corporation, Milford, CT, USA). Two columns were used: a Phenomenex Luna C18 column (250 × 10 mm, 10 μm; flow rate: 2 mL/min; Phenomenex) and a Phenomenex Luna Phenyl-Hexyl column (250 × 10 mm, 10 μm; flow rate: 2 mL/min; Phenomenex). Thin-layer chromatography (TLC) employed Merck precoated silica gel F254 plates and RP-C18 F254s plates. Spots were detected after TLC under UV light or by heating after spraying with anisaldehyde-sulfuric acid.

### 3.2. Plant Materials

*E. arvense* specimens were collected in June 2021 from Jecheon, Chungcheongbuk-do, Republic of Korea. A voucher specimen (HIMH-2113) was identified and authenticated by Dr. Hye-Ryen Na at the Northeastern Asia Biodiversity Institute, located in Seoul 05677, Republic of Korea. The collected material has been deposited in the herbarium of the Nakdonggang National Institute of Biological Resources in Sangju, Republic of Korea.

### 3.3. Extraction and Isolation

The aerial parts of *E. arvense* (1.2 kg) were dried at 35–45 °C in a plant drying oven for one week. Then, dried *E. arvense* was chopped and extracted using 80% ethanol (10 L) three times. The extract was condensed using a rotary evaporator to obtain a green crude extract (192.5 g). An appropriate amount (11.8 g) of crude extract was taken out, and reversed-phase Sep-Pak was conducted to divide the extract into a MeOH soluble fraction (7.5 g) and a CH_2_Cl_2_ soluble fraction. The MeOH soluble fraction was applied to Diaion HP-20 with distilled water to eliminate the sugar-rich part, and then an extract was gained by using MeOH as the mobile phase. The resultant extract (1.2 g) was separated into four fractions (M1–M4) via normal-phase silica MPLC using a gradient solvent system of CH_2_Cl_2_-MeOH (100:0–1:1, *v*/*v*). Fraction M2 (218.9 mg) was subjected to semi-preparative reversed-phase HPLC with 40% MeOH/H_2_O isocratic solvent system to isolate compounds **1** (3.4 mg, *t*_R_ = 14.0 min), **2** (0.5 mg, *t*_R_ = 27.2 min), **3** (2.5 mg, *t*_R_ = 18.9 min), and **4** (12.0 mg, *t*_R_ = 34.8 min). Fraction M3 (255.5 mg) was subjected to semi-preparative reversed-phase HPLC with 30% MeOH/H_2_O isocratic solvent system to isolate compounds **5** (2.2 mg, *t*_R_ = 17.9 min), **6** (0.6 mg, *t*_R_ = 35.8 min), and subfractions M35 (16.0 mg, *t*_R_ = 39.0 min) and M36 (3.0 mg, *t*_R_ = 46.2 min). Subfraction M35 (16.0 mg) was further subjected to semi-preparative reversed-phase HPLC with a 35% MeOH/H_2_O isocratic solvent system to isolate compound **7** (1.0 mg, *t*_R_ = 26.0 min). Subfraction M36 (3.0 mg) was further subjected to semi-preparative reversed-phase HPLC with a 35% MeOH/H_2_O isocratic solvent system to isolate compounds **8** (1.2 mg, *t*_R_ = 16.5 min) and **9** (0.8 mg, *t*_R_ = 22.3 min).

### 3.4. Chemicals and Reagents

Phosphate-buffered saline (PBS), Dulbecco’s modified essential medium (DMEM), fetal bovine serum (FBS), antibiotics (10,000 U/mL penicillin/10,000 μg/mL streptomycin), and 0.25% trypsin-EDTA were purchased from Gibco Life Technologies (Grand Island, NY, USA). PowerUp^TM^ SYBR Master Mix, Halt^TM^ protease and phosphatase inhibitor cocktails, and other reagents for quantitative real-time polymerase chain reaction (qRT-PCR) were obtained from Thermo Scientific Pierce (Waltham, MA, USA). Enzyme-linked immunosorbent assay (ELISA) kits and recombinant human TNFα and IFNγ were sourced from R&D Systems (Minneapolis, MN, USA). Primary antibodies, including anti-p-nuclear factor (NF)-κB p65, anti-p-c-Jun (AP-1), anti-p-IκB-α, anti-p-signal transducer and activator of transcription (STAT)-1, and anti-p-p38, were purchased from Cell Signaling Technology, Inc. (Beverly, MA, USA). Other primary antibodies were obtained from Santa Cruz Biotechnology (Santa Cruz, CA, USA). Equipment, chemicals, and horse radish peroxidase-conjugated secondary antibodies were sourced from Bio-Rad (Hercules, CA, USA). Other chemicals, including 3-(4,5-dimethyl-2-thiazolyl)-2,5-diphenyl-2H-tetrazolium bromide (MTT), were purchased from Sigma-Aldrich (St. Louis, MO, USA).

### 3.5. Cell Culture and Sample Treatment

The HaCaT cell line (Cell Line Service, Eppelheim, Germany), a human keratinocyte, was cultured and maintained in DMEM supplemented with 10% (*v*/*v*) FBS and 1% antibiotics, hereinafter referred to as the growth medium. The cells were incubated at 37 °C in a humidified incubator containing 5% CO_2_. Sub-culturing was performed when the cells reached approximately 80% confluence. For the experiments, cells were treated with the samples for 2 h and then continuously stimulated with or without TNFα/IFNγ (5 ng/mL each). Cells were allowed to grow to approximately 70–80% confluence to investigate cellular signaling pathways and were then subjected to starvation. This was achieved by replacing the existing growth media with DMEM containing 0.5% (*v*/*v*) FBS and 1% (*v*/*v*) antibiotics for 16 h.

### 3.6. Cell Viability

Cytotoxicity of compounds was assessed in HaCaT cells using an MTT assay with slight modifications based on a previous study [58]. Briefly, cells were seeded in 96-well culture plates at a density of 1.5 × 10^4^ cells per well and incubated for 24 h. They were then treated with different concentrations of compounds ranging from 0 to 80 μM, while some were simultaneously stimulated with TNFα/IFNγ. Following a 24-h incubation, the preexisting growth medium was replaced with a fresh growth medium containing MTT at a concentration of 0.5 mg/mL. The cells were further incubated for 1 h. Afterward, the supernatant was discarded, and dimethyl sulfoxide was added to each well. Absorbance measurements were taken at 570 nm. Cell viability was calculated using the following formula:

Cell viability (%): (A_sample_/A_control_) × 100

where A_sample_ and A_control_ are the absorbance of respective samples and negative-control, respectively.

### 3.7. ELISA and qRT-PCR

Production and mRNA levels of pro-inflammatory chemokines and cytokines were assessed using commercial ELISA kits and qRT-PCR analysis, respectively [1]. HaCaT cells were plated in 60 mm culture dishes at a density of 3.5 × 10^5^ cells per dish and incubated for 48 h. They were then treated with each compound at a concentration of 40 μM and induced with TNFα/IFNγ. After 20 h of incubation, the supernatant was collected for ELISA. Cells were washed with ice-cold PBS, and cellular total RNA was extracted using an RNeasy mini kit (Qiagen, Hilden, Germany). The obtained total RNA (2 μg) was synthesized into cDNA with a RevertAidTM first-strand cDNA synthesis kit (Thermo Fisher Scientific). PCR mixtures (20 μL) consisted of 100 ng of cDNA, 400 μM of human-specific primers, SYBR Green Master Mix, and nuclease-free water. Amplification was performed using a QuantStudio 6 Flex instrument (Thermo Fisher Scientific) according to the manufacturer’s instructions. Single-product amplification was confirmed by analyzing the melting curve. Relative mRNA expression levels were calculated using the delta-delta Cq (ΔΔCq) method, with glyceraldehyde 3-phosphate dehydrogenase (GAPDH) serving as the internal control gene. The sequence of specific primers used in the qRT-PCR analysis is presented in Table 1.

### 3.8. Western Blotting

Western blot analysis was conducted to determine the protein expression levels of inflammation-associated factors [58]. HaCaT cells were plated in 60 mm culture dishes at a density of 3.5 × 10^5^ cells per dish and incubated for 48 h. Afterward, the cells were starved for 16 h. Subsequently, the cells were treated with 40 μM of the compounds for 2 h and continuously stimulated with TNFα/IFNγ for 10 min. Following this treatment, the cells were washed three times with ice-cold PBS. Then, they were lysed in Pro-Prep protein extraction buffer (iNtRON Biotechnology, Gyeonggi-do, Republic of Korea) containing protease and phosphatase inhibitors. The cellular lysates underwent sonication (1 Amp; pulse-on, 5 s; pulse-off, 5 s) for a total period of 30 s and were then incubated for an additional 30 min in an ice bath. Supernatants were collected by centrifugation (15,000× *g*, 30 min, 4 °C), and the protein content was measured using a DC protein assay kit (Bio-Rad). Equal amounts of cellular proteins (ranging from 15 to 25 μg) were sequentially separated using 10% SDS-PAGE, transferred onto a PVDF (0.2 μm) membrane, blocked with 5% non-fat milk, probed with primary antibodies (1:2000), and subsequently incubated with secondary antibodies (1:10,000). After incubation with enhanced chemiluminescence for 2 min, each protein band was detected using an iBrightTM CL750 Imaging instrument (Invitrogen, Carlsbad, CA, USA). Densitometry analysis of each band was performed using Image J software version 1.8.0. (NIH, Bethesda, MD, USA).

### 3.9. Statistical Analysis

The results are presented as the mean ± standard deviation (SD) from independent experiments performed in triplicates. Statistical analysis was carried out using IBM SPSS for Windows version 18.0 (SPSS Inc., Chicago, IL, USA). The one-tailed, one-way analysis of variance (ANOVA) was utilized to analyze statistical differences between multiple groups. This was followed by Tukey’s post-hoc test. *p*-values less than 0.05 were considered statistically significant.

## 4. Conclusions

This study unveiled the anti-atopic potential of *E. arvense* by elucidating the phytochemical profiles of the ethanolic extract from *E. arvense* aerial parts and exploring their anti-inflammatory properties in TNFα/IFNγ-induced keratinocytes. A total of nine secondary metabolites were isolated, including two flavonoid glycosides (**1** and **4**), a megastigmane glycoside (**2**), a hexenyl-glycoside (**3**), a phenylpropanoid glycoside (**5**), lignan glycosides (**6** and **8**), and two caffeoylshikimic acids (**7** and **9**). Their structures were determined through a comprehensive chemical analysis involving liquid chromatography, NMR, HR-ESI-MS, and ECD spectroscopy. Notably, (*Z*)-3-hexenyl *β*-D-glucopyranoside (compound **3**) and lignan glycosides (compounds **6** and **8**) exhibited significant anti-inflammatory effects. They achieved this by inhibiting the expression of inflammation-associated chemokines and cytokines and mitigating the activation of pro-inflammatory transcription factors, including NF-κB, AP-1, and STAT1. While further investigations are needed to elucidate the cellular mechanisms and conduct in vivo studies, the findings from this study suggest that *E. arvense* has the potential to alleviate aberrant inflammatory responses in dermal tissues. Overall, *E. arvense* holds promise for the development of prophylactic and therapeutic products targeting inflammatory skin diseases.

## Figures and Tables

**Table 1:** Human-specific PCR primer sequences used in this experiment.

Gene	Primer Sequence
*CCL17*	Forward: 5′-CAGCTCGAGGGACCAATGTG-3′
	Reverse: 5′-CCTGCCCTGCACAGTTACAA-3′
*CCL5*	Forward: 5′-CAGTCGTCTTTGTCACCCGA-3′
	Reverse: 5′-TCTTCTCTGGGTTGGCACAC-3′
*CCL22*	Forward: 5′-ACTCCTGGTTGTCCTCGTC-3′
	Reverse: 5′-GACGTAATCACGGCAGCAGA-3′
*CCL2*	Forward: 5′-AATCAATGCCCCAGTCACCT-3′
	Reverse: 5′-CTTCTTTGGGACACTTGCTGC-3′
*IL1B*	Forward: 5′-CAGCTACGAATCTCCGACCAC-3′
	Reverse: 5′-GGCAGGGAACCAGCATCTTC-3′
*IL6*	Forward: 5′-TTCGGTCCAGTTGCCTTCTC-3′
	Reverse: 5′-TCTTCTCCTGGGGGTACTGG-3′
*CXCL8*	Forward: 5′-TGTCTGGACCCCAAGGAAAAC-3′
	Reverse: 5′-TGGCATCTTCACTGATTCTTGG-3′
*G3PD*	Forward: 5′-GAAGGTGAAGGTCGGAGTC-3′
	Reverse: 5′-GAAGATGGTGATGGGATTTC-3′

*CCL17*, thymus and activation-regulated chemokine (TARC); *CCL5*, regulated on activation, normal T cell expressed and secreted (RANTES); *CCL22*, macrophage-derived chemokine (MDC); *CCL2*, monocyte chemoattractant protein (MCP)-1; *IL1B*, interleukin-1β; *IL6*, interleukin-6; *CXCL8*, C-X-C motif chemokine ligand 8; *G3PD*, glyceraldehyde-3-phosphate dehydrogenase (GAPDH).

## Data Availability

Data is contained within the article.

## Supplementary Materials

The following supporting information can be downloaded at: https://www.mdpi.com/article/10.3390/ph16101478/s1, Figure S1: The HR-ESIMS data of Compound **1**; Figure S2: The ^1^H NMR spectrum of Compound **1** (DMSO-*d_6_*, 850 MHz); Figure S3: The HR-ESIMS data of Compound **2**; Figure S4: The ^1^H NMR spectrum of Compound **2** (DMSO-*d_6_*, 850 MHz); Figure S5: The HR-ESIMS data of Compound **3**; Figure S6: The ^1^H NMR spectrum of Compound **3** (DMSO-*d_6_*, 850 MHz); Figure S7: The HR-ESIMS data of Compound **4**; Figure S8: The ^1^H NMR spectrum of Compound **4** (DMSO-*d_6_*, 850 MHz); Figure S9: The HR-ESIMS data of Compound **5**; Figure S10: The ^1^H NMR spectrum of Compound **5** (CD_3_OD, 850 MHz); Figure S11: The HR-ESIMS data of Compound **6**; Figure S12: The ^1^H NMR spectrum of Compound **6** (CD_3_OD, 850 MHz); Figure S13: The HR-ESIMS data of Compound **7**; Figure S14: The ^1^H NMR spectrum of Compound **7** (CD_3_OD, 850 MHz); Figure S15: The HR-ESIMS data of Compound **8**; Figure S16: The ^1^H NMR spectrum of Compound **8** (CD_3_OD, 850 MHz); Figure S17: The HR-ESIMS data of Compound **9**; Figure S18: The ^1^H NMR spectrum of Compound **9** (CD_3_OD, 850 MHz).

## References

[1] Kim J.K., Choi E., Hong Y.H., Kim H., Jang Y.-J., Lee J.S., Choung E.S., Woo B.Y., Hong Y.D., Lee S. (2021). Syk/NF-κB-targeted anti-inflammatory activity of *Melicope accedens* (Blume) TG Hartley methanol extract. J. Ethnopharmacol..

[2] Hossen M.J., Amin A., Fu X.-Q., Chou J.-Y., Wu J.-Y., Wang X.-Q., Chen Y.-J., Wu Y., Li J., Yin C.-L. (2021). The anti-inflammatory effects of an ethanolic extract of the rhizome of *Atractylodes lancea*, involves Akt/NF-κB signaling pathway inhibition. J. Ethnopharmacol..

[3] Yu H.-S., Lee N.-K., Choi A.-J., Choe J.-S., Bae C.H., Paik H.-D. (2019). Anti-inflammatory potential of probiotic strain *Weissella cibaria* JW15 isolated from kimchi through regulation of NF-κB and MAPKs pathways in LPS-induced RAW 264.7 cells. J. Microbiol. Biotechnol..

[4] Yang J.-H., Hwang Y.-H., Gu M.-J., Cho W.-K., Ma J.Y. (2015). Ethanol extracts of *Sanguisorba officinalis* L. suppress TNF-α/IFN-γ-induced pro-inflammatory chemokine production in HaCaT cells. Phytomedicine.

[5] Jin H., Kumar L., Mathias C., Zurakowski D., Oettgen H., Gorelik L., Geha R. (2009). Toll-like receptor 2 is important for the TH1 response to cutaneous sensitization. J. Allergy Clin. Immunol..

[6] Choi J.H., Jin S.W., Park B.H., Kim H.G., Khanal T., Han H.J., Hwang Y.P., Choi J.M., Chung Y.C., Hwang S.K. (2013). Cultivated ginseng inhibits 2, 4-dinitrochlorobenzene-induced atopic dermatitis-like skin lesions in NC/Nga mice and TNF-α/IFN-γ-induced TARC activation in HaCaT cells. Food Chem. Toxicol..

[7] Trautmann A., Akdis M., Kleemann D., Altznauer F., Simon H.-U., Graeve T., Noll M., Bröcker E.-B., Blaser K., Akdis C.A. (2000). T cell–mediated Fas-induced keratinocyte apoptosis plays a key pathogenetic role in eczematous dermatitis. J. Clin. Investig..

[8] Lee J.-H., Lim J.-Y., Jo E.H., Noh H.M., Park S., Park M.C., Kim D.-K. (2020). Chijabyukpi-Tang Inhibits pro-inflammatory cytokines and chemokines via the Nrf2/HO-1 signaling pathway in TNF-α/IFN-γ-stimulated HaCaT cells and ameliorates 2, 4-dinitrochlorobenzene-induced atopic dermatitis-like skin lesions in mice. Front. Pharmacol..

[9] Avena-Woods C. (2017). Overview of atopic dermatitis. Am. J. Manag. Care.

[10] Guttman-Yassky E., Nograles K.E., Krueger J.G. (2011). Contrasting pathogenesis of atopic dermatitis and psoriasis—Part I: Clinical and pathologic concepts. J. Allergy Clin. Immunol..

[11] Nedoszytko B., Sokołowska-Wojdyło M., Ruckemann-Dziurdzińska K., Roszkiewicz J., Nowicki R. (2014). Chemokines and cytokines network in the pathogenesis of the inflammatory skin diseases: Atopic dermatitis, psoriasis and skin mastocytosis. Postepy Dermatol Alergol.

[12] Lee Y., Choi H.K., N’deh K.P.U., Choi Y.-J., Fan M., Kim E.-k., Chung K.-H., An J.H. (2020). Inhibitory effect of *Centella asiatica* extract on DNCB-induced atopic dermatitis in HaCaT cells and BALB/c mice. Nutrients.

[13] Peng W., Novak N. (2015). Pathogenesis of atopic dermatitis. Clin. Exp. Allergy.

[14] Wollenberg A., Oranje A., Deleuran M., Simon D., Szalai Z., Kunz B., Svensson A., Barbarot S., Von Kobyletzki L., Taieb A. (2016). ETFAD/EADV Eczema task force 2015 position paper on diagnosis and treatment of atopic dermatitis in adult and paediatric patients. J. Eur. Acad. Dermatol. Venereol..

[15] Dawid-Pac R. (2013). Medicinal plants used in treatment of inflammatory skin diseases. Postepy Dermatol Alergol.

[16] Al-Snafi A.E. (2017). The pharmacology of *Equisetum arvense*-A review. IOSR J. Pharm..

[17] Sandhu N.S., Kaur S., Chopra D. (2010). *Equisetum arvense*: Pharmacology and phytochemistry-a review. Asian. J. Pharm. Clin. Res..

[18] Asgarpanah J., Roohi E. (2012). Phytochemistry and pharmacological properties of *Equisetum arvense* L.. J. Med. Plant Res..

[19] Gründemann C., Lengen K., Sauer B., Garcia-Käufer M., Zehl M., Huber R. (2014). *Equisetum arvense* (common horsetail) modulates the function of inflammatory immunocompetent cells. BMC Complement. Altern. Med..

[20] Shiba F., Miyauchi M., Chea C., Furusho H., Iwasaki S., Shimizu R., Ohta K., Nishihara T., Takata T. (2021). Anti-inflammatory effect of glycyrrhizin with *Equisetum arvense* extract. Odontology.

[21] Shiba F., Furusho H., Takata T., Shimizu R., Miyauchi M. (2022). *Equisetum arvense* inhibits alveolar bone destruction in a rat model with lipopolysaccharide (LPS)-induced periodontitis. Int. J. Dent..

[22] Mimica-Dukic N., Simin N., Cvejic J., Jovin E., Orcic D., Bozin B. (2008). Phenolic compounds in field horsetail (*Equisetum arvense* L.) as natural antioxidants. Molecules.

[23] Garcia D., Ramos A.J., Sanchis V., Marín S. (2013). *Equisetum arvense* hydro-alcoholic extract: Phenolic composition and antifungal and antimycotoxigenic effect against *Aspergillus flavus* and *Fusarium verticillioides* in stored maize. J. Sci. Food Agric..

[24] Kukrić Z., Topalić-Trivunović L., Pavičić S., Žabić M., Matoš S., Davidović A. (2013). Total phenolic content, antioxidant and antimicrobial activity of *Equisetum arvense* L.. Chem. Ind. Chem. Eng. Q..

[25] Steinborn C., Potterat O., Meyer U., Trittler R., Stadlbauer S., Huber R., Gründemann C. (2018). In vitro anti-inflammatory effects of *Equisetum arvense* are not solely mediated by silica. Planta Med..

[26] Lee B.S., So H.M., Kim S., Kim J.K., Kim J.-C., Kang D.-M., Ahn M.-J., Ko Y.-J., Kim K.H. (2022). Comparative evaluation of bioactive phytochemicals in *Spinacia oleracea* cultivated under greenhouse and open field conditions. Arch. Pharm. Res..

[27] Cho H., Kim K.H., Han S.H., Kim H.-J., Cho I.-H., Lee S. (2022). Structure determination of heishuixiecaoline A from *Valeriana fauriei* and its content from different cultivated regions by HPLC/PDA Analysis. Nat. Prod. Sci..

[28] Yu J.S., Jeong S.Y., Li C., Oh T., Kwon M., Ahn J.S., Ko S.-K., Ko Y.-J., Cao S., Kim K.H. (2022). New phenalenone derivatives from the Hawaiian volcanic soil-associated fungus *Penicillium herquei* FT729 and their inhibitory effects on indoleamine 2, 3-dioxygenase 1 (IDO1). Arch. Pharm. Res..

[29] Lee S.R., Lee B.S., Yu J.S., Kang H., Yoo M.J., Yi S.A., Han J.-W., Kim S., Kim J.K., Kim J.-C. (2022). Identification of anti-adipogenic withanolides from the roots of Indian ginseng (*Withania somnifera*). J. Ginseng Res..

[30] Lee K.H., Kim J.K., Yu J.S., Jeong S.Y., Choi J.H., Kim J.-C., Ko Y.-J., Kim S.-H., Kim K.H. (2021). Ginkwanghols A and B, osteogenic coumaric acid-aliphatic alcohol hybrids from the leaves of *Ginkgo biloba*. Arch. Pharm. Res..

[31] Wan C., Yu Y., Zhou S., Tian S., Cao S. (2011). Isolation and identification of phenolic compounds from *Gynura divaricata* leaves. Pharmacogn. Mag..

[32] Woo K.W., Lee K.R. (2013). Phytochemical constituents of *Allium victorialis* var. platyphyllum. Nat. Prod. Sci.

[33] Lee K.H., Choi S.U., Lee K.R. (2005). Sesquiterpenes from *Syneilesis palmata* and their cytotoxicity against human cancer cell lines in vitro. Arch. Pharm. Res..

[34] Zhou X.-J., Yan L.-L., Yin P.-P., Shi L.-L., Zhang J.-H., Liu Y.-J., Ma C. (2014). Structural characterisation and antioxidant activity evaluation of phenolic compounds from cold-pressed *Perilla frutescens* var. arguta seed flour. Food. Chem..

[35] Cui C.-B., Tezuka Y., Kikuchi T., Nakano H., Tamaoki T., Park J.-H. (1990). Constituents of a fern, *Davallia mariesii* Moore. I. Isolation and structures of davallialactone and a new flavanone glucuronide. Chem. Pharm. Bull..

[36] El Gamal A., Takeya K., Itokawa H., Halim A., Amer M., Saad H.-E. (1997). Lignan bis-glucosides from *Galium sinaicum*. Phytochemistry.

[37] Saito T., Yamane H., Murofushi N., Takahashi N., Phinney B.O. (1997). 4-O-caffeoylshikimic and 4-O-(p-coumaroyl) shikimic acids from the dwarf tree fern, *Dicksonia antarctica*. Biosci. Biotechnol. Biochem..

[38] Huo C., Liang H., Zhao Y., Wang B., Zhang Q. (2008). Neolignan glycosides from *Symplocos caudata*. Phytochemistry.

[39] Li X., Zhang Y., Yang L., Feng Y., Liu Y., Zeng X. (2012). Studies of phenolic acid constituents from the whole plant of *Sarcandra glabra*. Zhongyao Xinyao Yu Linchuang Yaoli.

[40] Veit M., Geiger H., Czygan F.-C., Markham K.R. (1990). Malonylated flavone 5-O-glucosides in the barren sprouts of *Equisetum arvense*. Phytochemistry.

[41] Francescato L.N., Debenedetti S.L., Schwanz T.G., Bassani V.L., Henriques A.T. (2013). Identification of phenolic compounds in *Equisetum giganteum* by LC–ESI-MS/MS and a new approach to total flavonoid quantification. Talanta.

[42] Wang Z., Tian Y., Sugimoto S., Yamano Y., Kawakami S., Otsuka H., Matsunami K. (2022). Four new glucosides from the aerial parts of *Equisetum sylvaticum*. J. Nat. Med..

[43] Kanchanapoom T., Otsuka H., Ruchirawat S. (2007). Megastigmane glucosides from *Equisetum debile* and *E. diffusum*. Chem. Pharm. Bull..

[44] Boeing T., Tafarelo Moreno K.G., Gasparotto Junior A., Mota da Silva L., de Souza P. (2021). Phytochemistry and pharmacology of the genus *Equisetum* (Equisetaceae): A narrative review of the species with therapeutic potential for kidney diseases. Evid. Based Complement. Alternat. Med..

[45] Yang J.-H., Yoo J.-M., Lee E., Lee B., Cho W.-K., Park K.-I., Ma J.Y. (2018). Anti-inflammatory effects of *Perillae Herba* ethanolic extract against TNF-α/IFN-γ-stimulated human keratinocyte HaCaT cells. J. Ethnopharmacol..

[46] Brunner P.M., Guttman-Yassky E., Leung D.Y. (2017). The immunology of atopic dermatitis and its reversibility with broad-spectrum and targeted therapies. J. Allergy Clin. Immunol..

[47] Albanesi C., Scarponi C., Giustizieri M.L., Girolomoni G. (2005). Keratinocytes in inflammatory skin diseases. Curr. Drug. Targets. Inflamm. Allergy.

[48] An E.-J., Kim Y., Lee S.-H., Choi S.-H., Chung W.S., Jang H.-J. (2020). Ophiopogonin D ameliorates DNCB-induced atopic dermatitis-like lesions in BALB/c mice and TNF-α-inflamed HaCaT cell. Biochem. Biophys. Res. Commun..

[49] Dorjsembe B., Nho C.W., Choi Y., Kim J.-C. (2022). Extract from black soybean cultivar a63 extract ameliorates atopic dermatitis-like skin inflammation in an oxazolone-induced murine model. Molecules.

[50] Kwon D.-J., Bae Y.-S., Ju S.M., Goh A.R., Youn G.S., Choi S.Y., Park J. (2012). Casuarinin suppresses TARC/CCL17 and MDC/CCL22 production via blockade of NF-κB and STAT1 activation in HaCaT cells. Biochem. Biophys. Res. Commun..

[51] Nakayama T., Hieshima K., Nagakubo D., Sato E., Nakayama M., Kawa K., Yoshie O. (2004). Selective induction of Th2-attracting chemokines CCL17 and CCL22 in human B cells by latent membrane protein 1 of Epstein-Barr virus. J. Virol..

[52] Thao N.P., Luyen B.T.T., Tai B.H., Cuong N.M., Kim Y.C., Van Minh C., Kim Y.H. (2015). Chemical constituents of *Miliusa balansae* leaves and inhibition of nitric oxide production in lipopolysaccharide-induced RAW 264.7 cells. Bioorg. Med. Chem. Lett..

[53] Wang C., Xiao Y., Yang B., Wang Z., Wu L., Su X., Brantner A., Kuang H., Wang Q. (2014). Isolation and screened neuroprotective active constituents from the roots and rhizomes of *Valeriana amurensis*. Fitoterapia.

[54] Ngan N.T.T., Quang T.H., Tai B.H., Song S.B., Lee D., Kim Y.H. (2012). Anti-inflammatory and PPAR transactivational effects of components from the stem bark of Ginkgo biloba. J. Agric. Food Chem..

[55] Nahar P.P., Driscoll M.V., Li L., Slitt A.L., Seeram N.P. (2014). Phenolic mediated anti-inflammatory properties of a maple syrup extract in RAW 264.7 murine macrophages. J. Funct. Foods.

[56] Cho J.Y., Kim A.R., Park M.H. (2001). Lignans from the rhizomes of Coptis japonica differentially act as anti-inflammatory principles. Planta Med..

[57] Gao Q., Yang M., Zuo Z. (2018). Overview of the anti-inflammatory effects, pharmacokinetic properties and clinical efficacies of arctigenin and arctiin from *Arctium lappa* L.. Acta Pharmacol. Sin..

[58] Yu H.-S., Kim W.-J., Bae W.-Y., Lee N.-K., Paik H.-D. (2020). *Inula britannica* inhibits adipogenesis of 3T3-L1 preadipocytes via modulation of mitotic clonal expansion involving ERK 1/2 and Akt signaling pathways. Nutrients.

