# Supplementary material for: Phytochemical Investigation of Equisetum arvense and Evaluation of Their Anti-Inflammatory Potential in TNFα/INFγ-Stimulated Keratinocytes
Source: Pharmaceuticals (Basel). 2023 Oct 16;16(10):1478. doi: 10.3390/ph16101478 (PMC10609812; doi:10.3390/ph16101478)

Supporting Information

Phytochemical Investigation of *Equisetum arvense* and Evaluation of their Anti-inflammatory Potential in TNFα/INFγ-stimulated keratinocytes

Se Yun Jeong [1,†], Hyung-Seok Yu [2,†], Moon-Jin Ra [3], Sang-Mi Jung [3], Jeong-Nam Yu [4], Jin-Chul Kim [2,5,*] and Ki Hyun Kim [1,*]

[1]School of Pharmacy, Sungkyunkwan University, Suwon 16419, Republic of Korea; dlawktkark@naver.com (S.Y.J.)
[2]Natural Product Research Institute, Korea Institute of Science and Technology, Gangneung 25451, Republic of Korea; hsyu1207@kist.re.kr (H.S.Y.)
[3]Hongcheon Institute of Medicinal Herb, Hongcheon-gun, Gangwon-do 25142, Republic of Korea; ramj90@himh.re.kr (M.J.R.); sgmo77@naver.com (S.M.J.)
[4]Nakdonggang National Institute of Biological Resources, Sangju, Gyeongsangbuk-do 37242, Republic of Korea; susia000@nnibr.re.kr (J.N.Y.)
[5]Division of Bio-Medical Science and Technology, University of Science and Technology (UST), Daejeon 34113, Republic of Korea
*Correspondence: jckim@kist.re.kr (J.C.K.); khkim83@skku.edu (K.H.K.); +82-31-290-7700 (K.H.K.)
[†]These authors contributed equally to this study

Supporting Information Contents:

Figure S1. The HR-ESI-MS data of **1**……S3
Figure S2. The ^{1}H NMR spectrum of **1** (DMSO-d_6 850 MHz) ……S4
Figure S3. The HR-ESI-MS data of **2**……S5
Figure S4. The ^{1}H NMR spectrum of **2** (DMSO-d_6, 850 MHz) ……S6
Figure S5. The HR-ESI-MS data of **3**……S7
Figure S6. The ^{1}H NMR spectrum of **3** (DMSO-d_6, 850 MHz) ……S8
Figure S7. The HR-ESI-MS data of **4**……S9
Figure S8. The ^{1}H NMR spectrum of **4** (DMSO-d_6, 850 MHz) ……S10
Figure S9. The HR-ESI-MS data of **5**……S11
Figure S10. The ^{1}H NMR spectrum of **5** (CD_3OD, 850 MHz) ……S12
Figure S11. The HR-ESI-MS data of **6**……S13
Figure S12. The ^{1}H NMR spectrum of **6** (CD_3OD, 850 MHz) ……S14
Figure S13. The HR-ESI-MS data of **7**……S15
Figure S14. The ^{1}H NMR spectrum of **7** (CD_3OD, 850 MHz) ……S16
Figure S15. The HR-ESI-MS data of **8**……S17
Figure S16. The ^{1}H NMR spectrum of **8** (CD_3OD, 850 MHz) ……S18
Figure S17. The HR-ESI-MS data of **9**……S19
Figure S18. The ^{1}H NMR spectrum of **9** (CD_3OD, 850 MHz) ……S20

Figure S1. The HR-ESIMS data of **1**

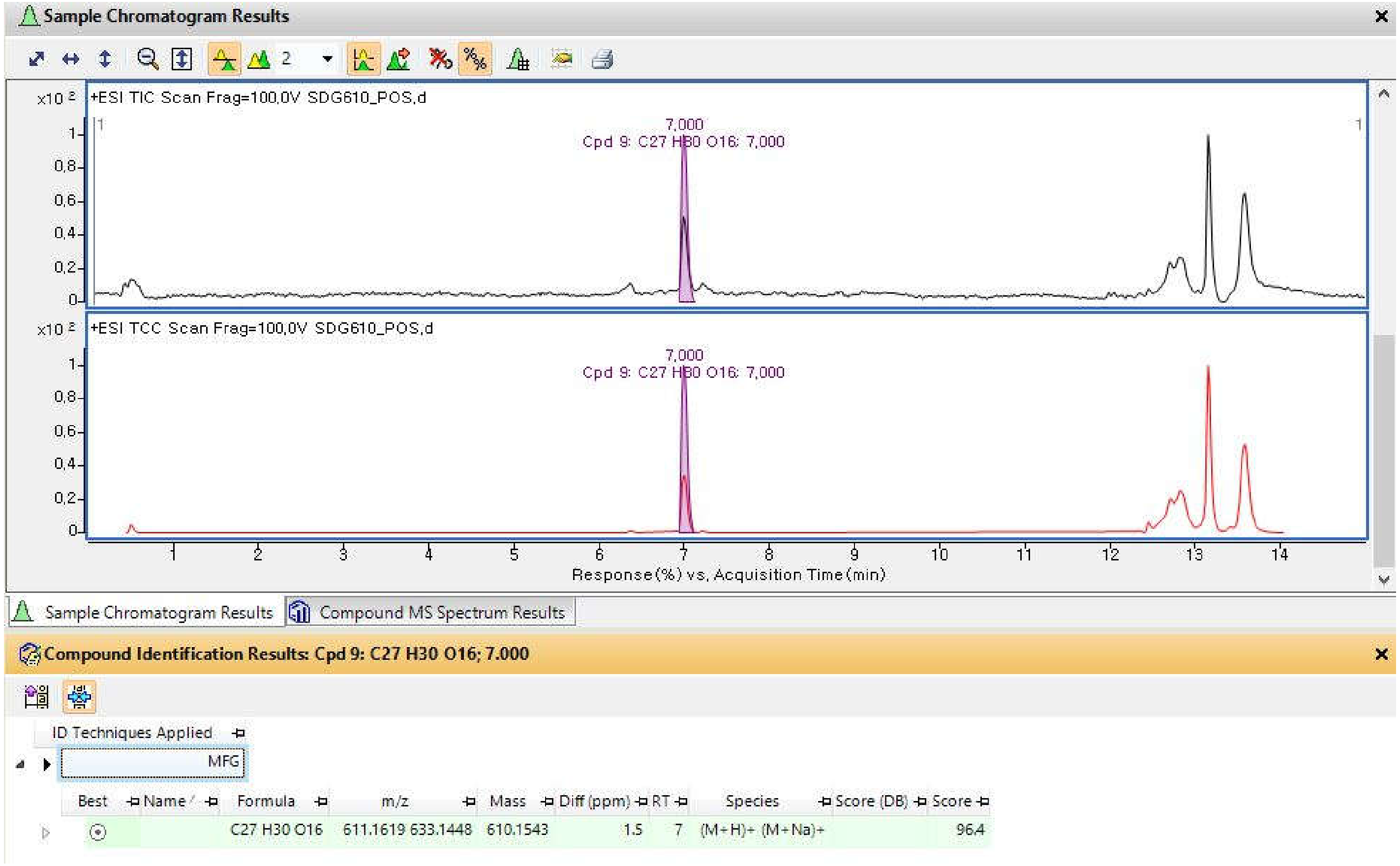

Figure S2. The ^{1}H NMR spectrum of **1** (DMSO-d_6, 850 MHz)

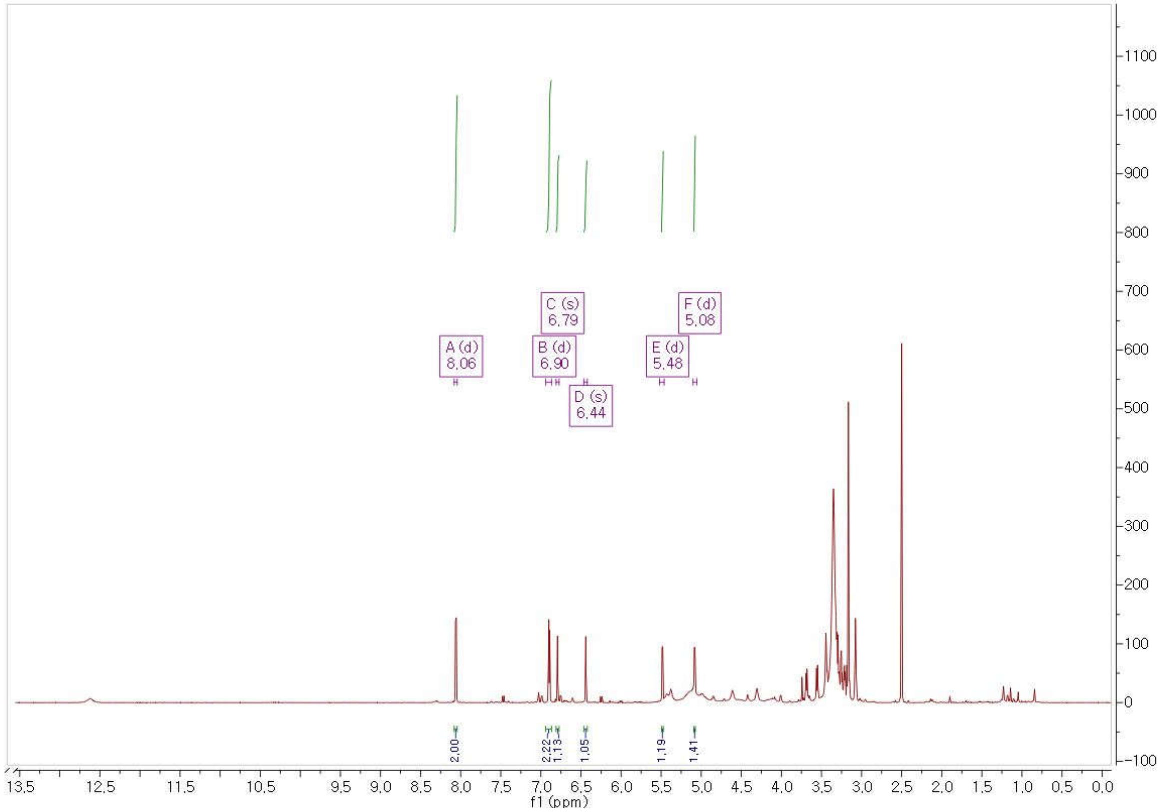

Figure S3. The HR-ESIMS data of **2**

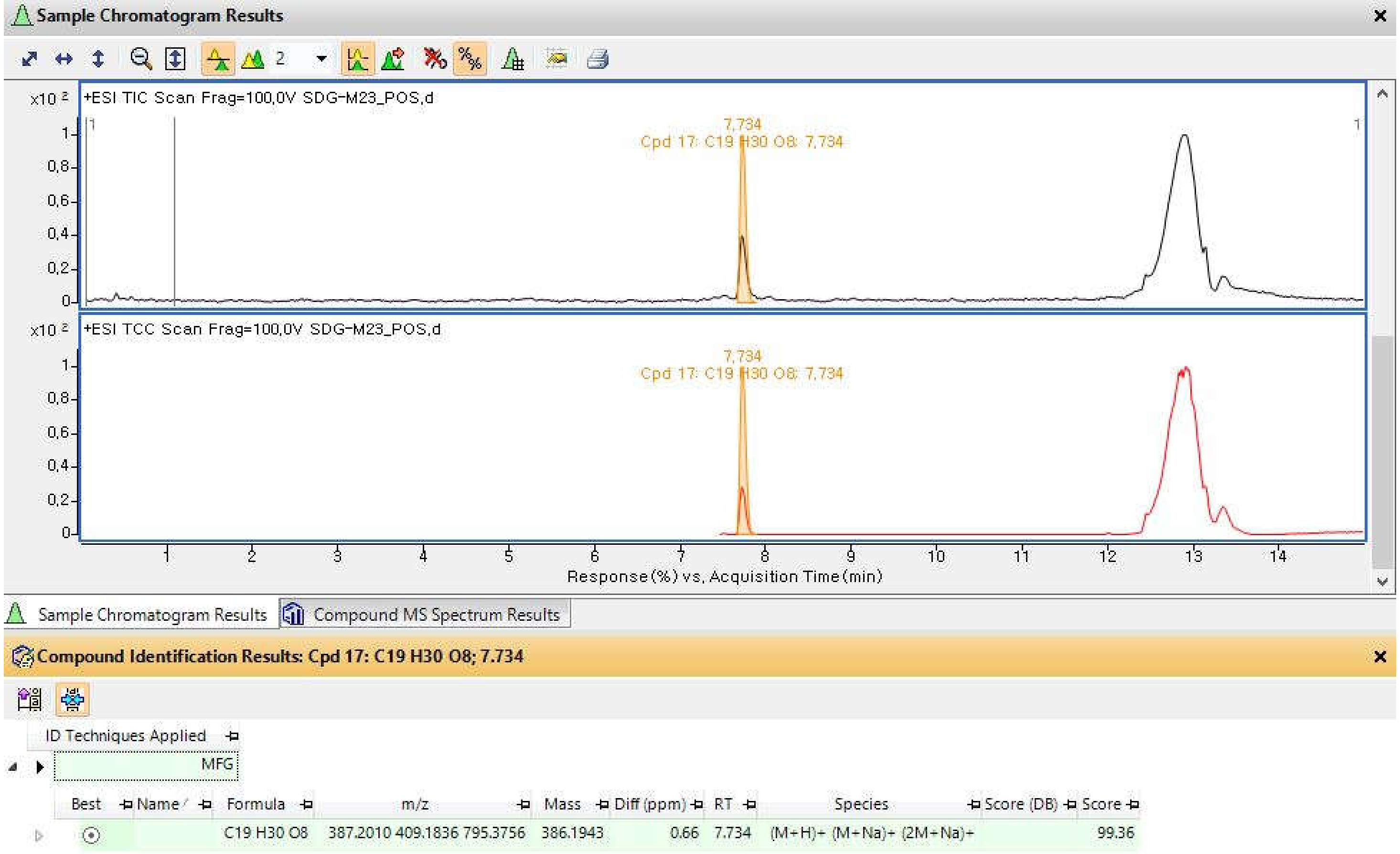

Figure S4. The ^{1}H NMR spectrum of **2** (DMSO-d_6, 850 MHz)

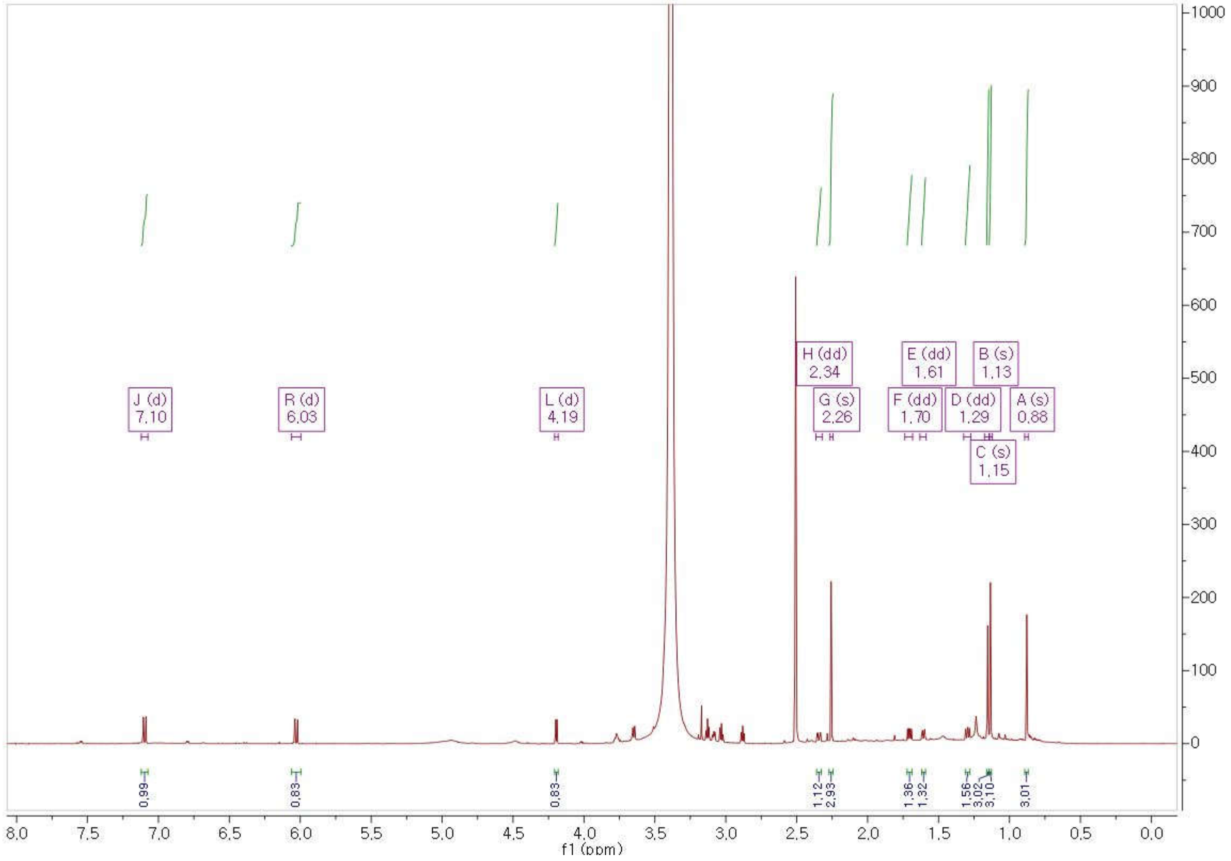

Figure S5. The HR-ESIMS data of **3**

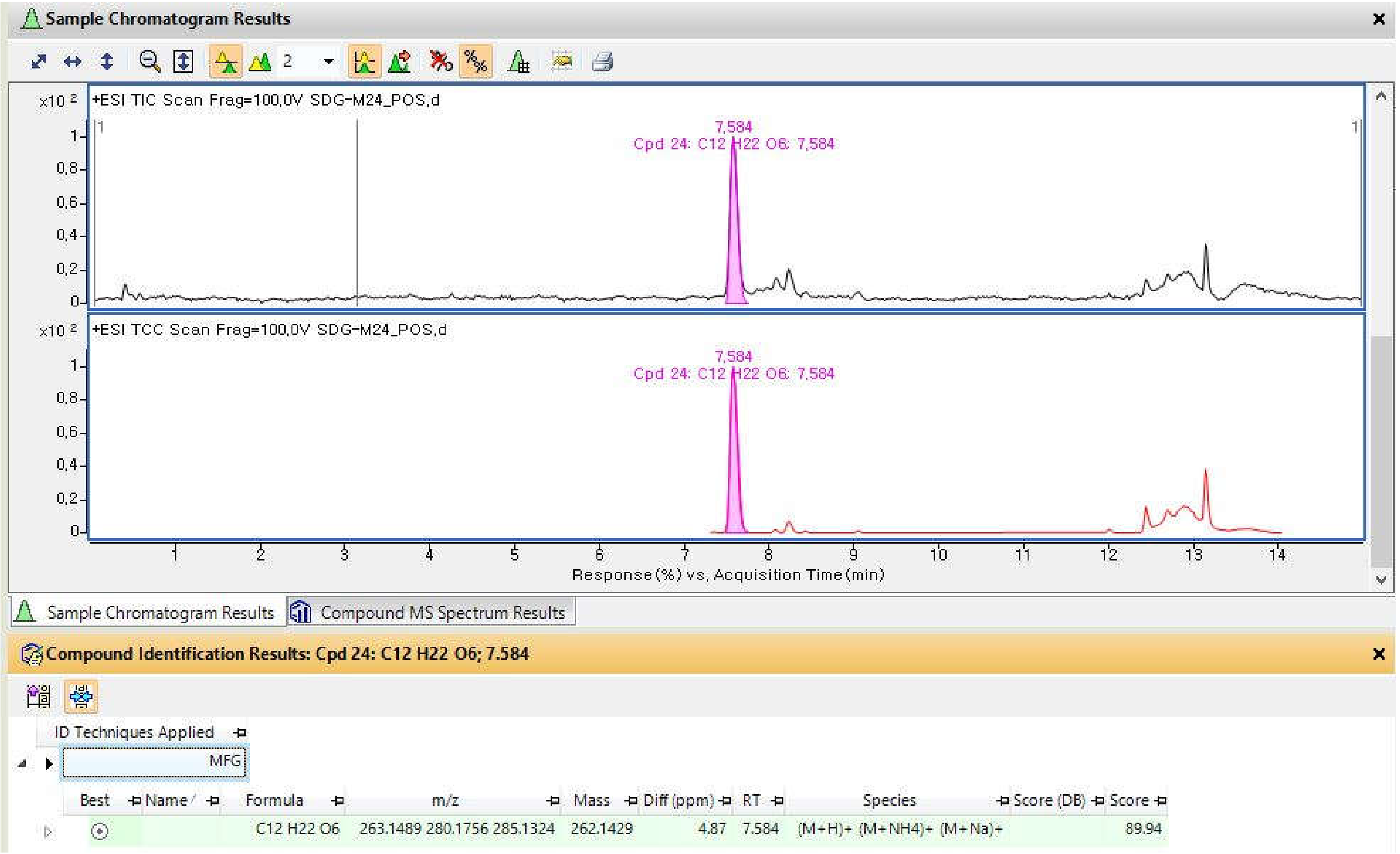

Figure S6. The ^{1}H NMR spectrum of **3** (DMSO-d_6, 850 MHz)

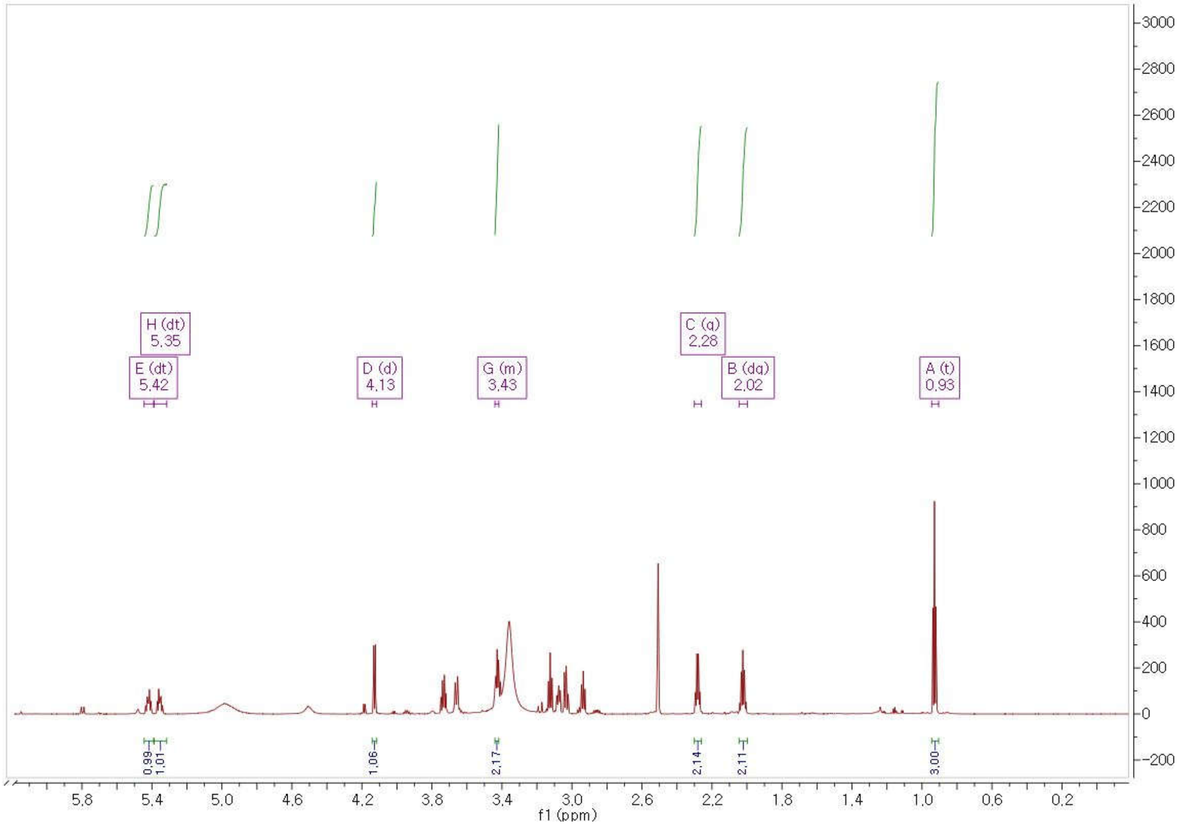

Figure S7. The HR-ESIMS data of **4**

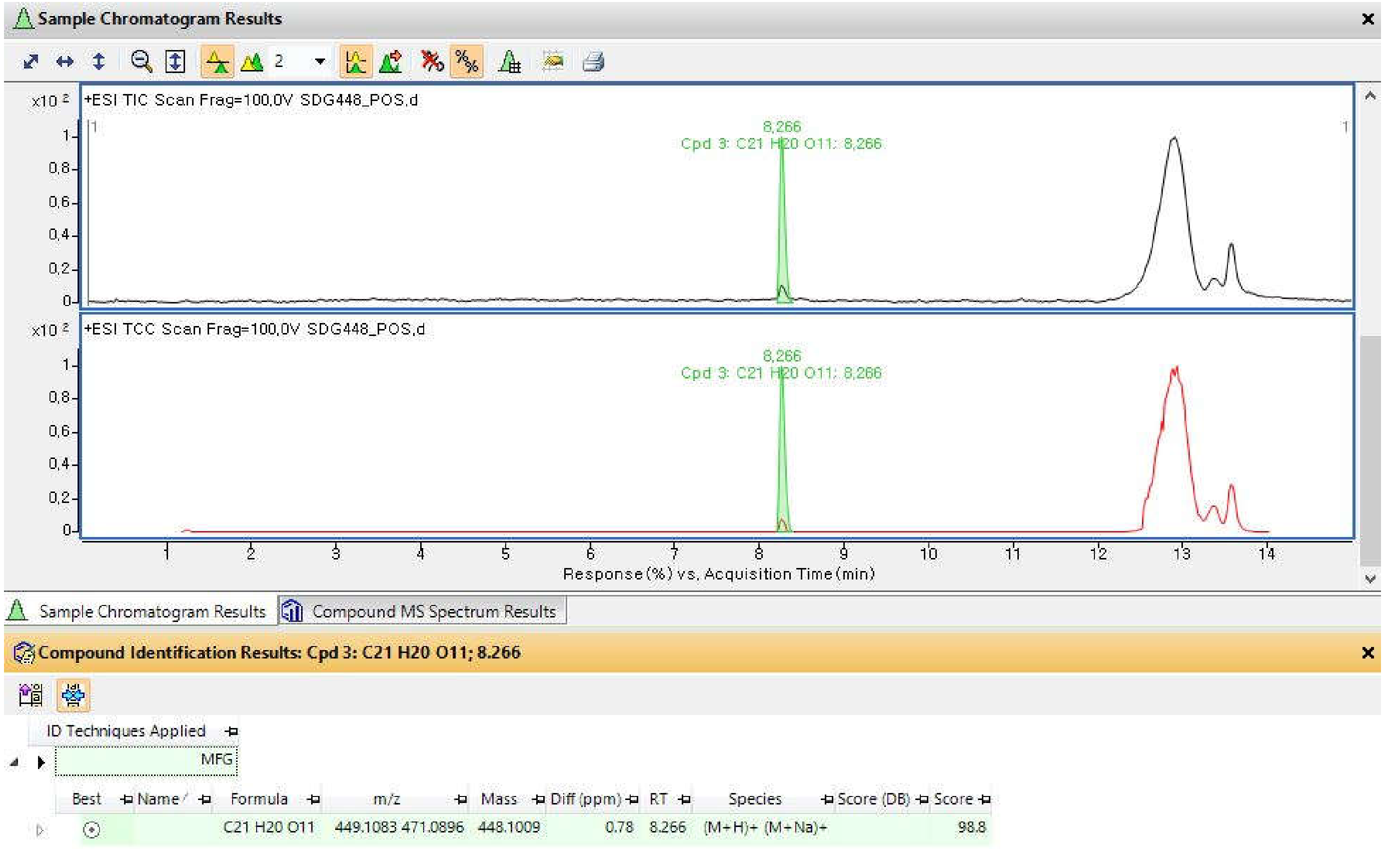

Figure S8. The ^{1}H NMR spectrum of **4** (DMSO-d_6, 850 MHz)

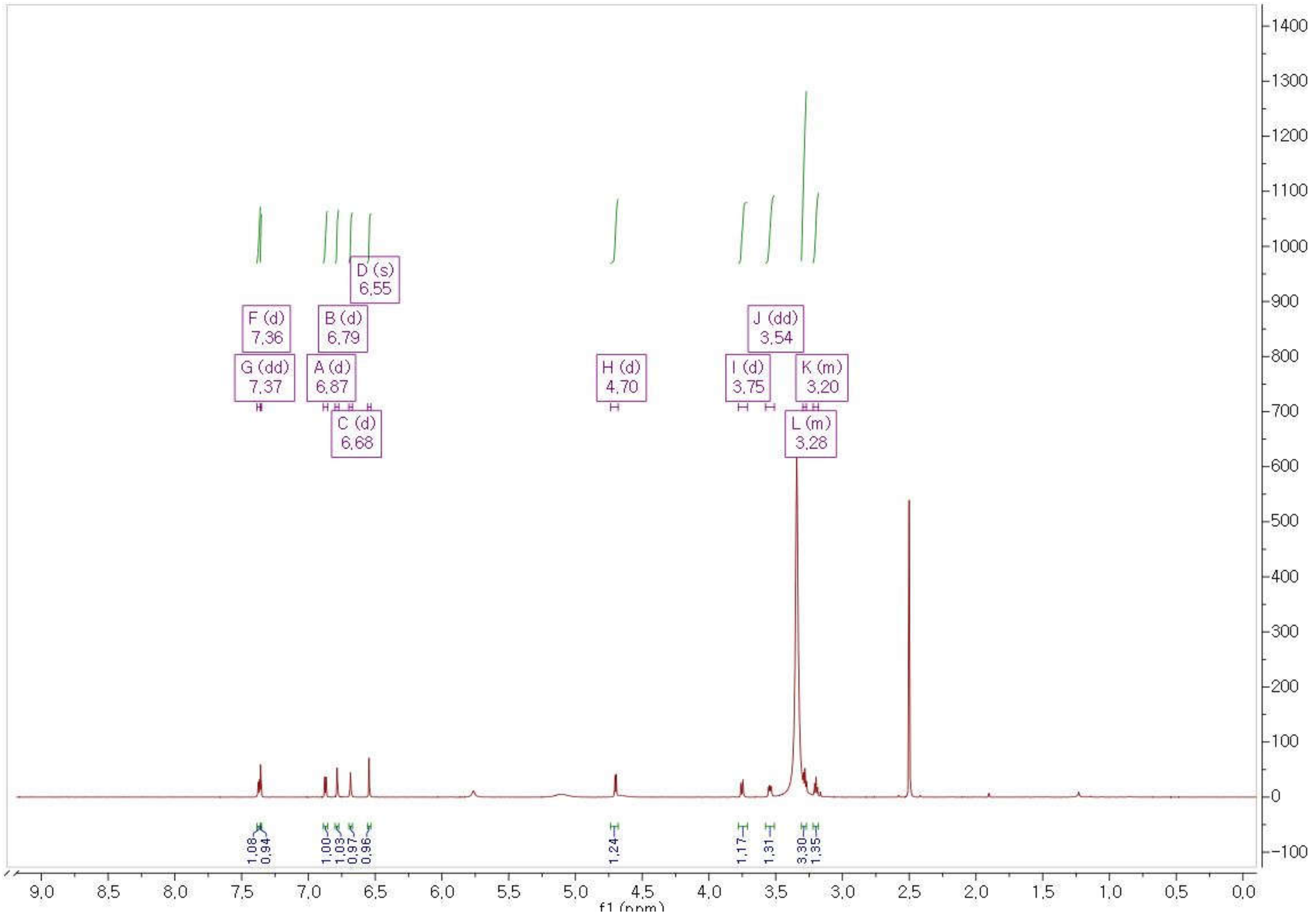

Figure S9. The HR-ESIMS data of **5**

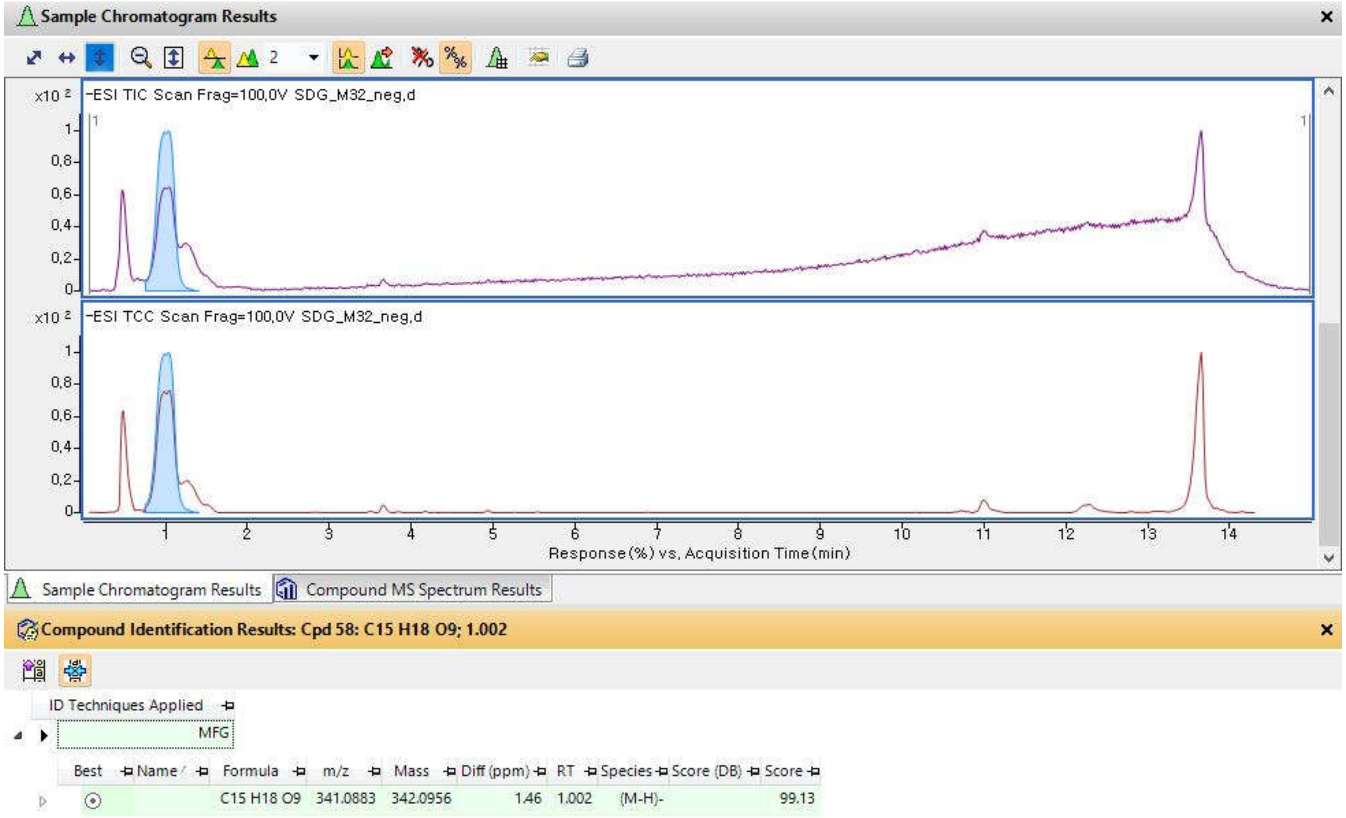

Figure S10. The ^{1}H NMR spectrum of **5** (CD_3OD, 850 MHz)

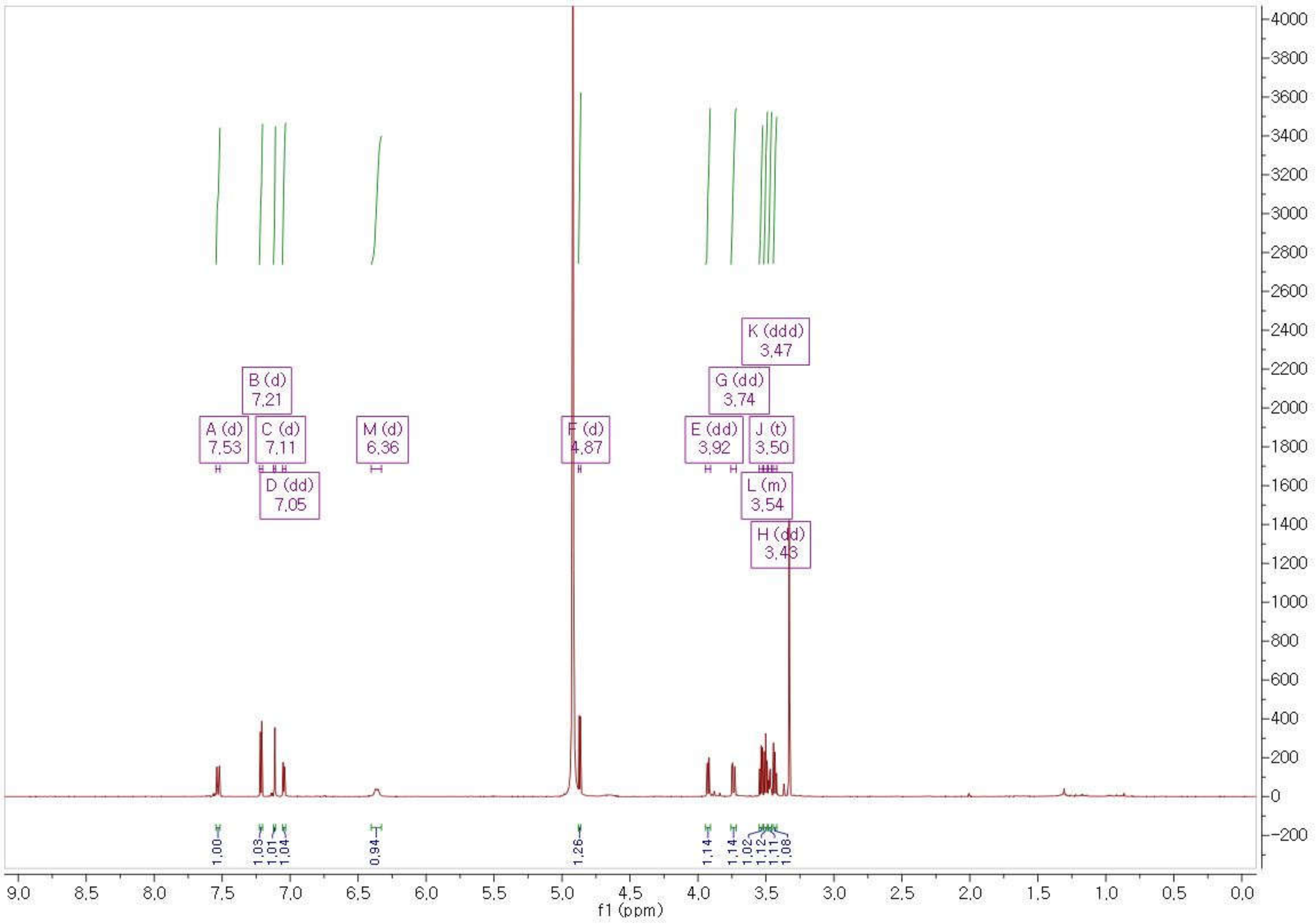

Figure S11. The HR-ESIMS data of **6**

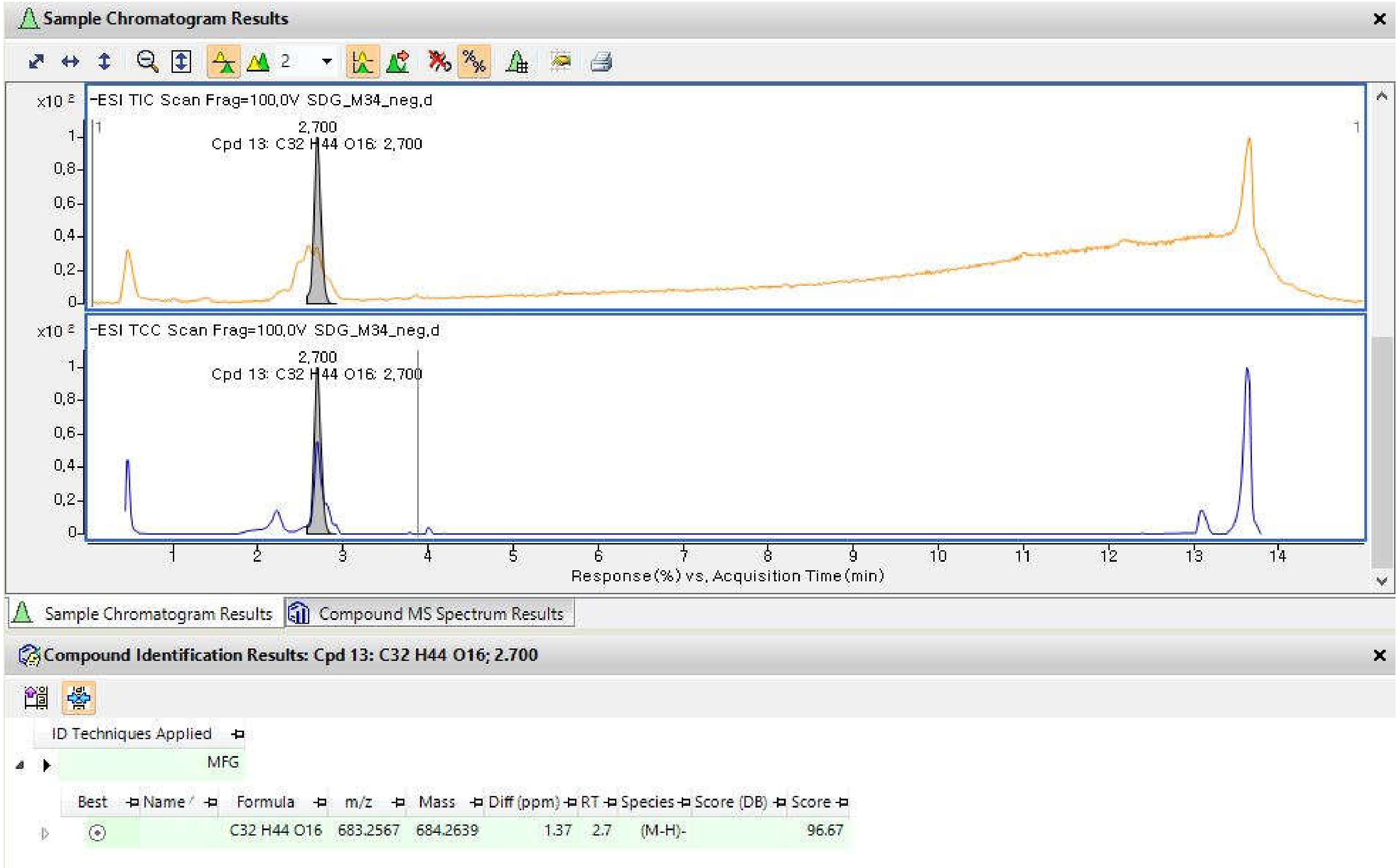

Figure S12. The ^{1}H NMR spectrum of **6** (CD_3OD, 850 MHz)

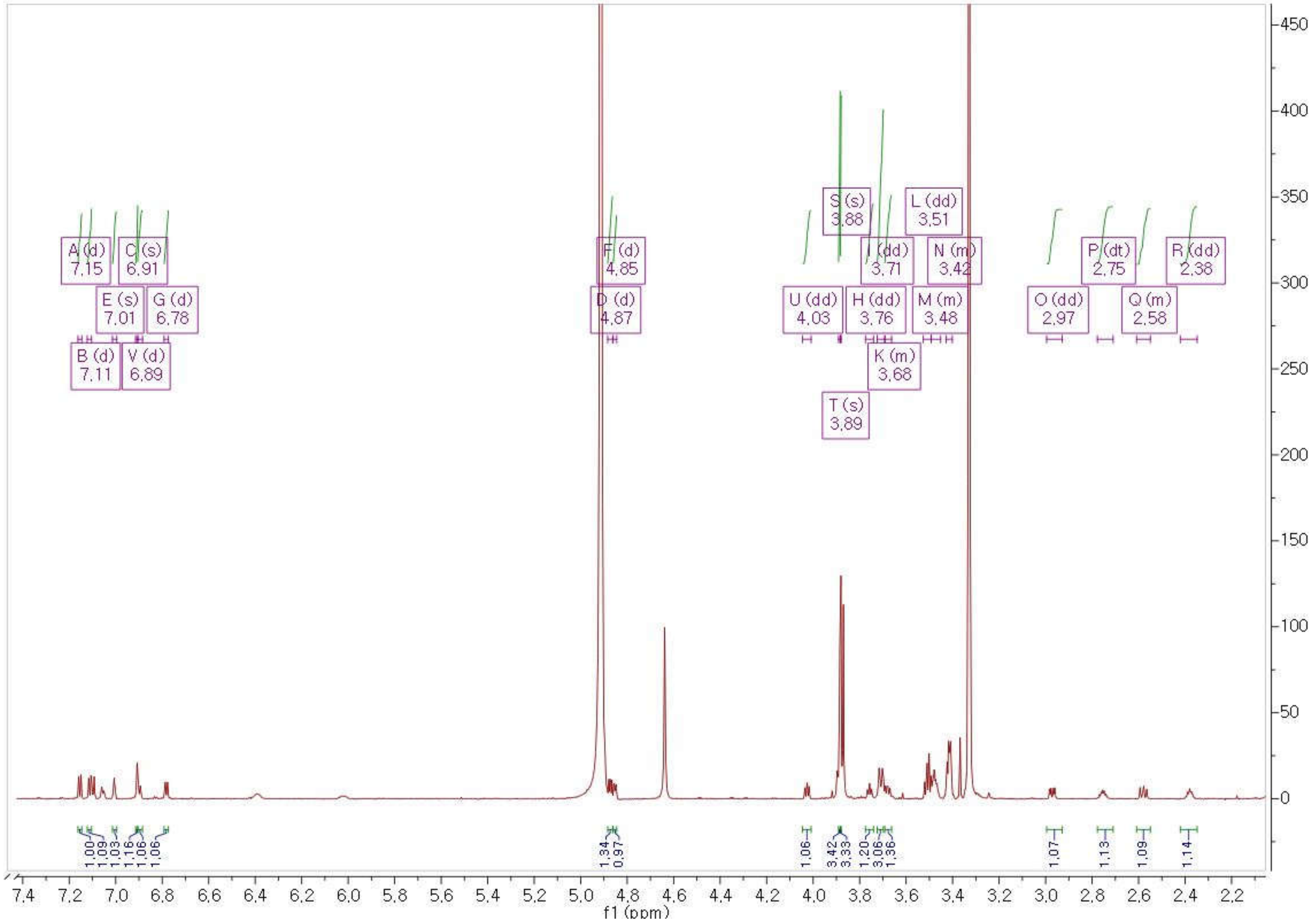

Figure S13. The HR-ESIMS data of **7**

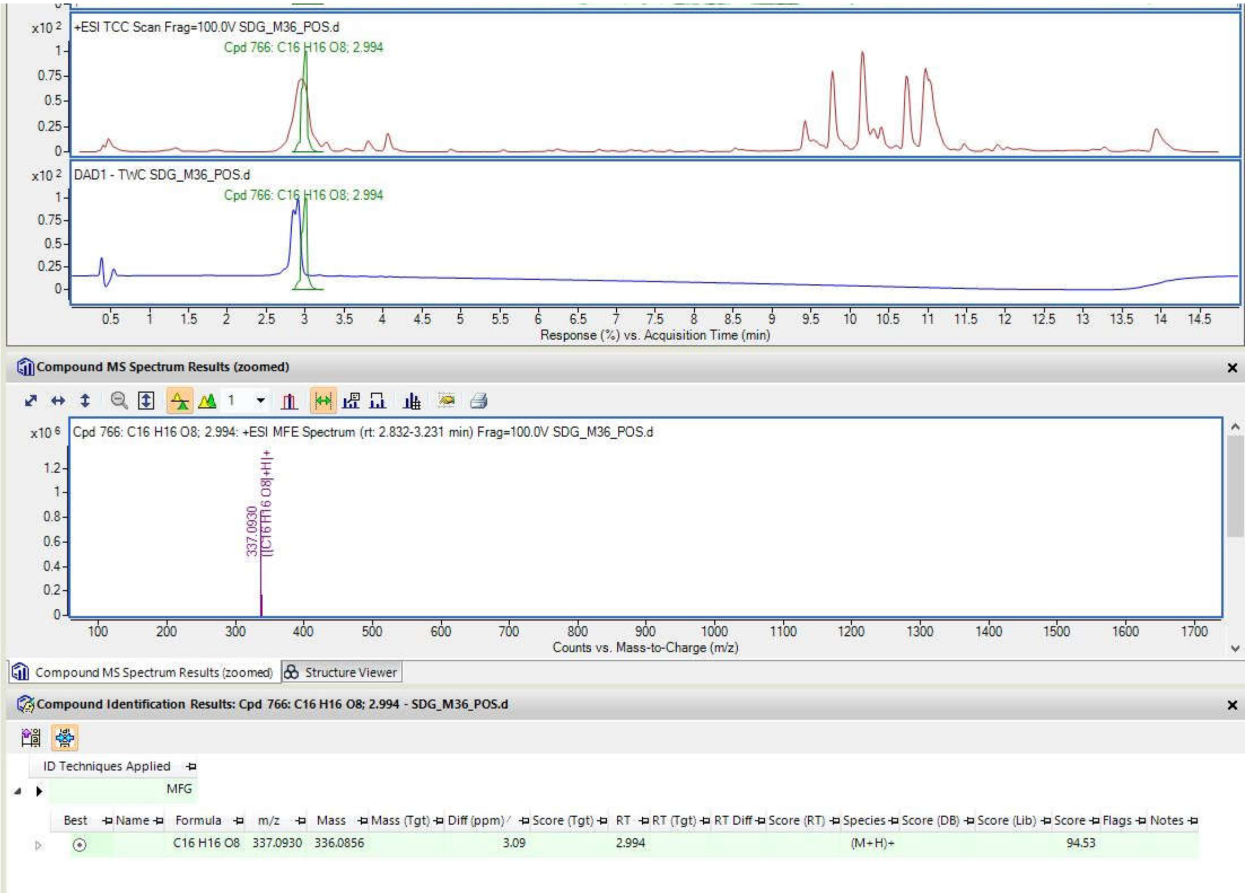

Figure S14. The ^{1}H NMR spectrum of **7** (CD_3OD, 850 MHz)

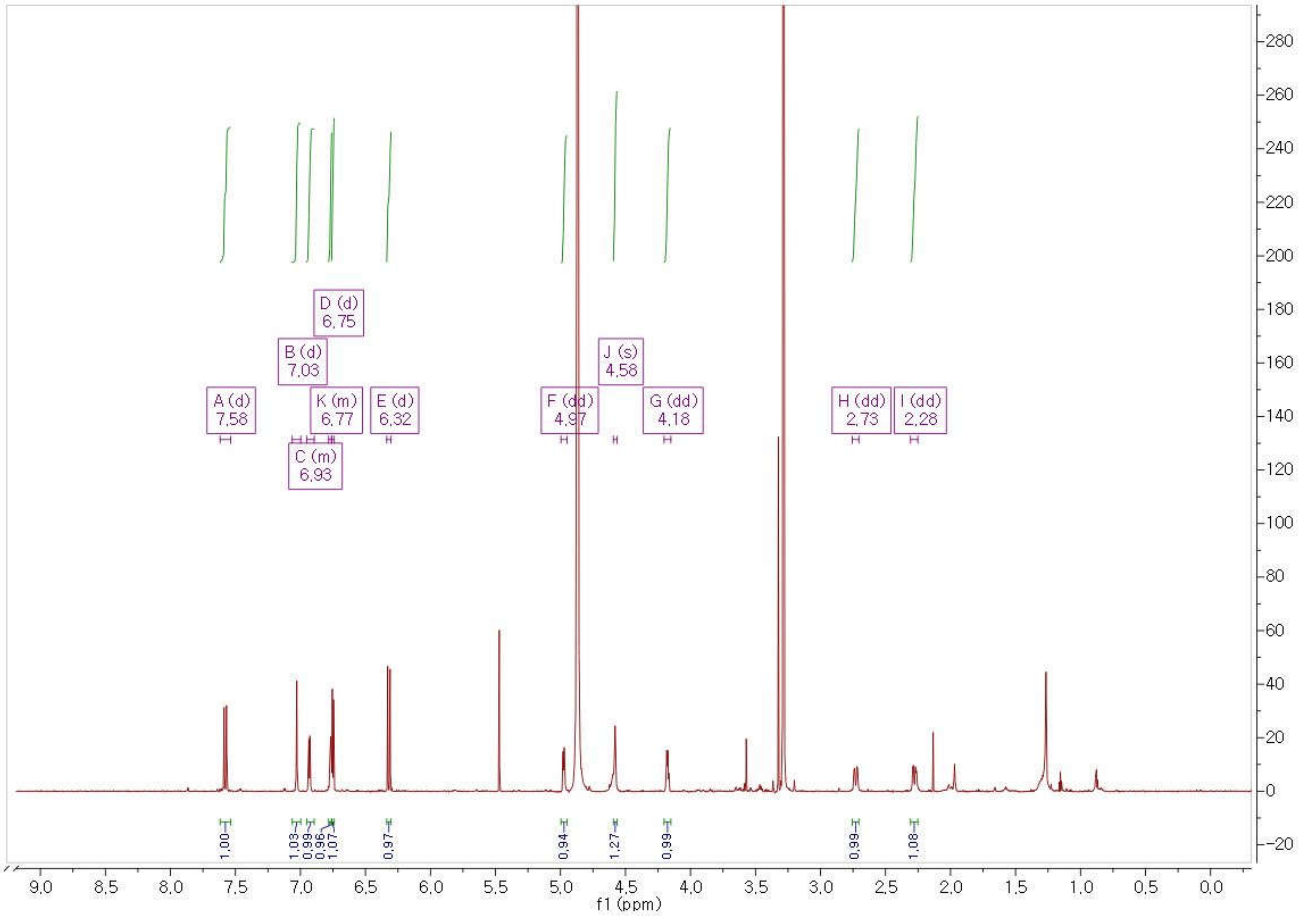

Figure S15. The HR-ESIMS data of **8**

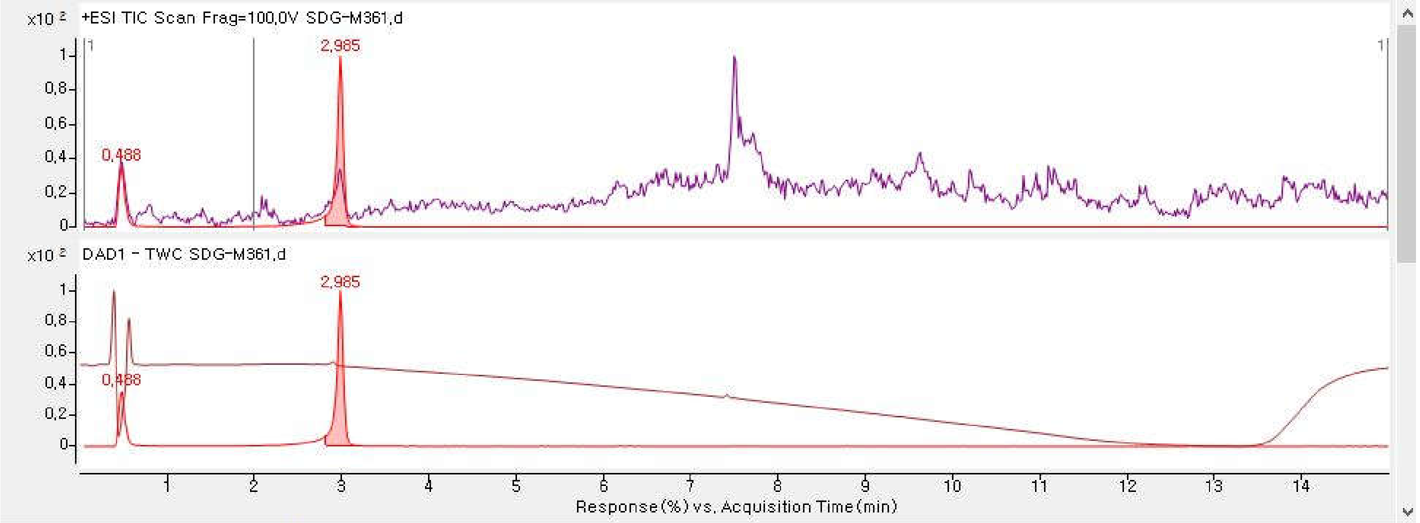

Sample Chromatogram Results | Compound MS Spectrum Results

Compound Identification Results: Cpd 1: C26 H36 O12; 2.985

ID Techniques Applied
FBF

Best	Name	Formula	m/z	Mass	Diff (ppm)	RT	Species	Score (DB)	Score
⊙		C26 H36 O12	558.2546 563.2106	540.2213	1.16	2.985	(M+NH4)+ (M+Na)+		97

Figure S16. The ^{1}H NMR spectrum of **8** (CD_3OD, 850 MHz)

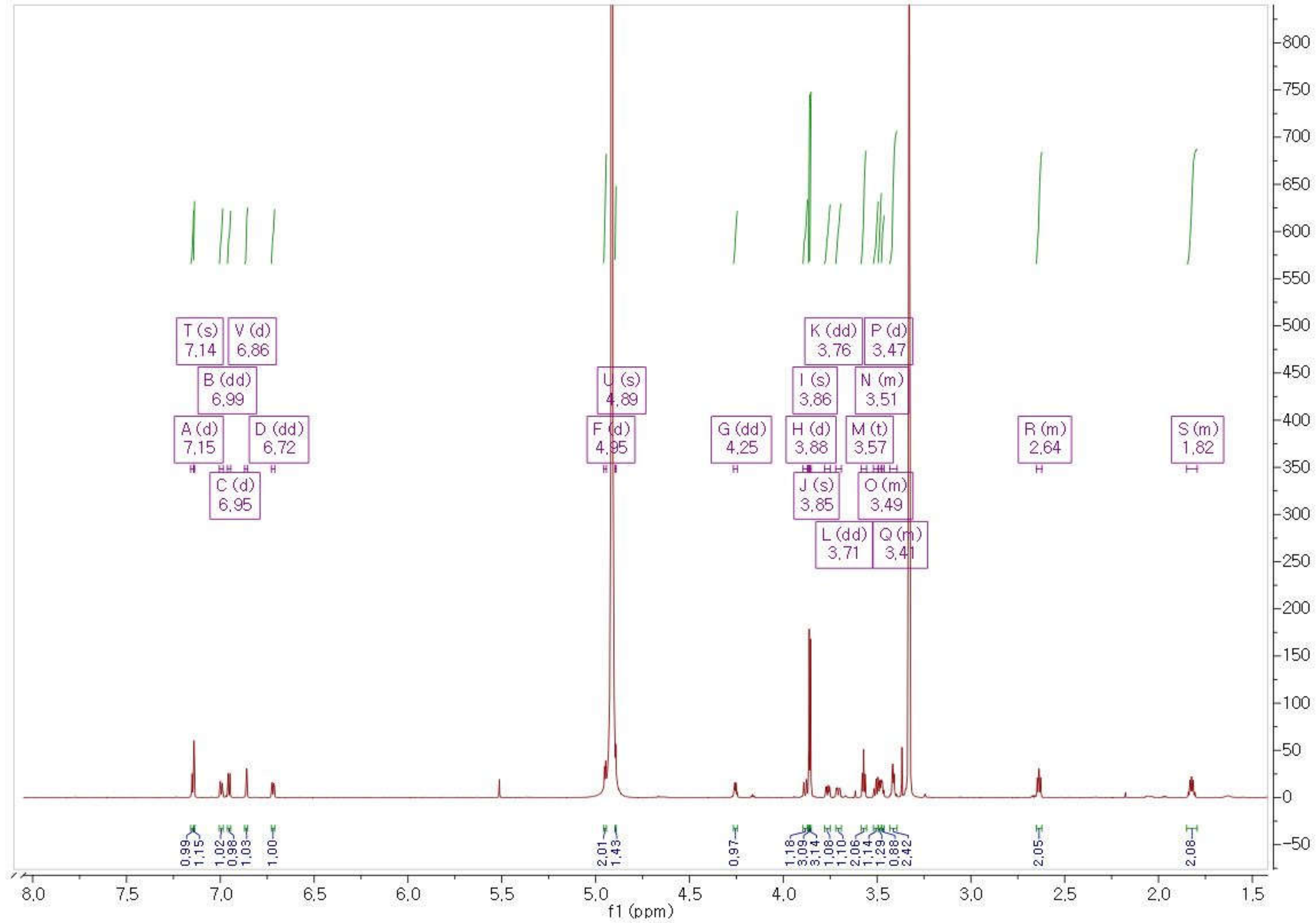

Figure S17. The HR-ESIMS data of **9**

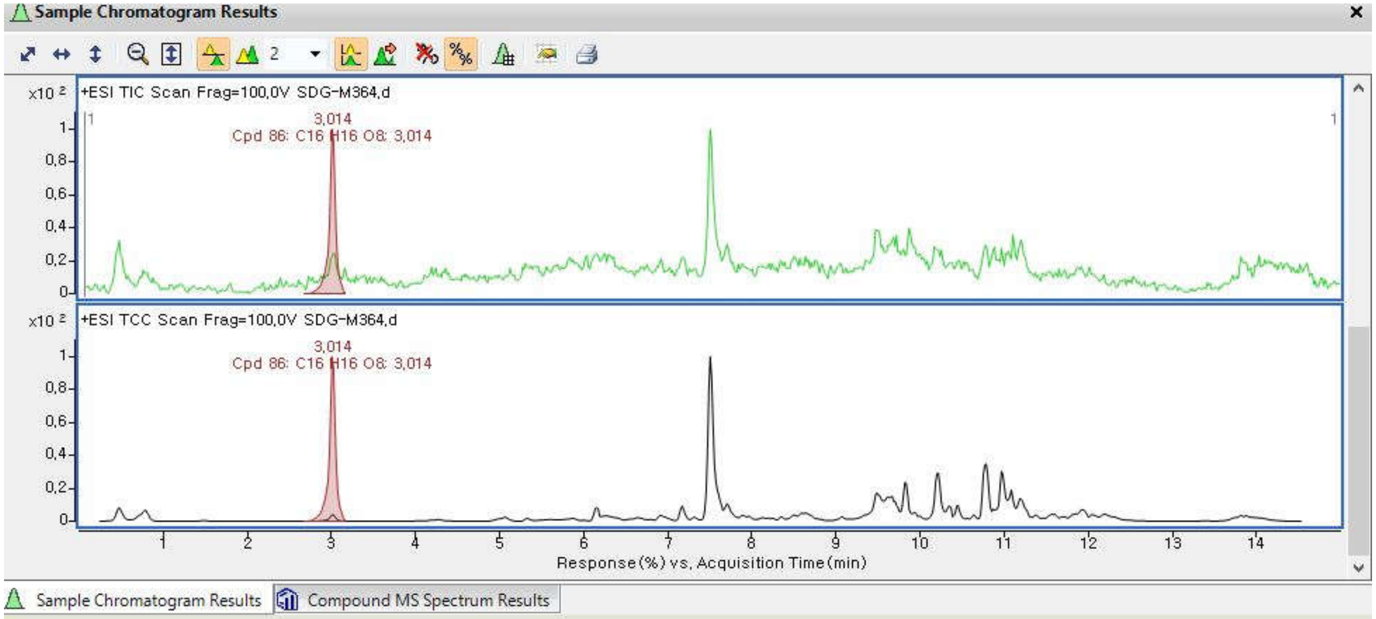

Compound Identification Results: Cpd 86: C16 H16 O8; 3.014

ID Techniques Applied: MFG

Best	Name	Formula	m/z	Mass	Diff (ppm)	RT	Species	Score (DB)	Score
⊙		C16 H16 O8	337.0920 359.0737	336.0848	0.98	3.014	(M+H)+ (M+Na)+		97.79

Figure S18. The ^{1}H NMR spectrum of **9** (CD_3OD, 850 MHz)

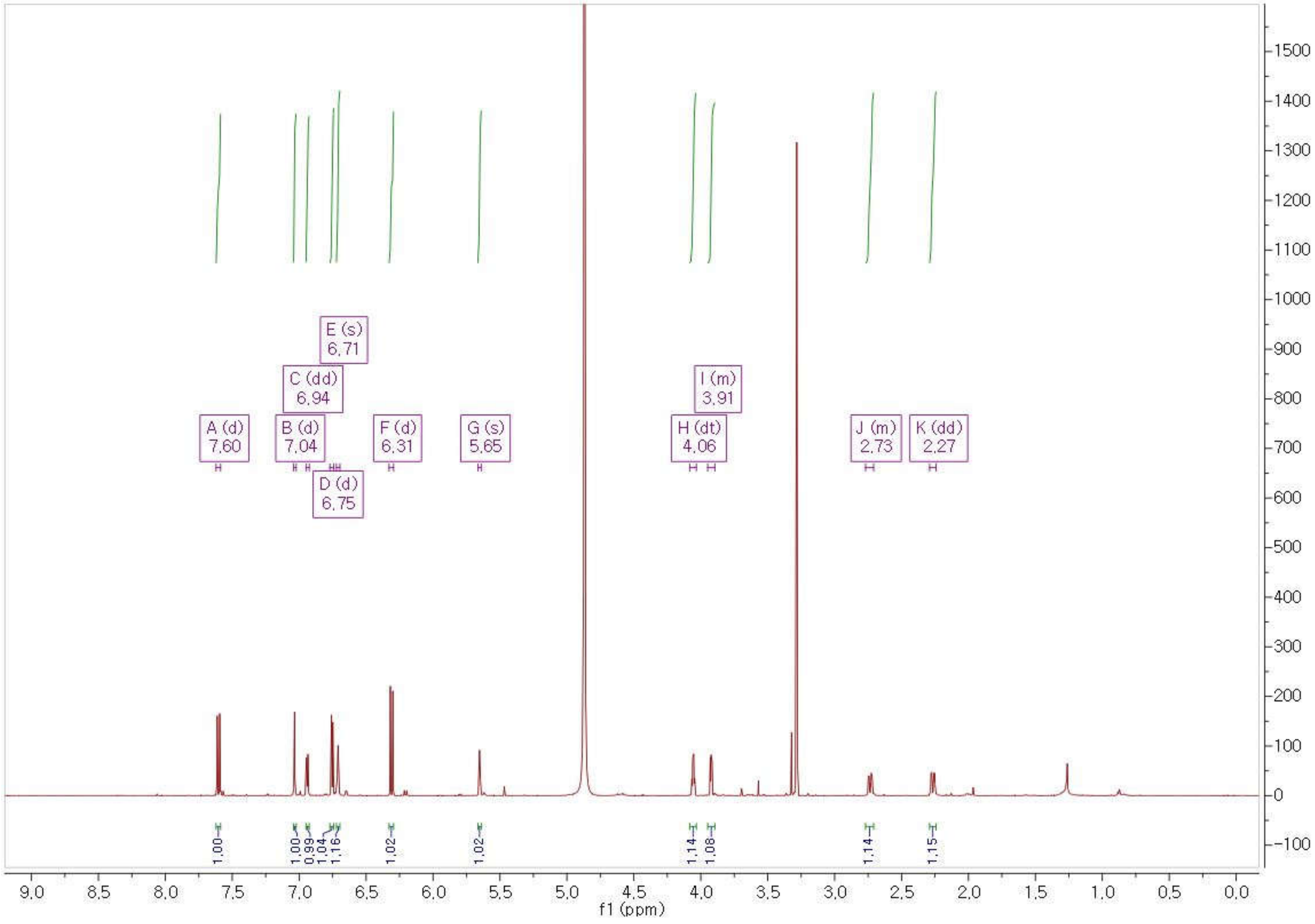

Supplement: Supplementary file 1 [file pharmaceuticals-16-01478-s001.zip › pharmaceuticals-2635977-supplementary.pdf]
